# Prevalence and transmission of HIV-1 drug resistance mutations among patients with treatment failure and newly diagnosed people in Liangshan Prefecture, China, in 2021–2023

**DOI:** 10.3389/fpubh.2025.1550121

**Published:** 2025-03-12

**Authors:** Rong Pei, Yulian Zhang, Chunnong Jike, Gang Yu, Ling Su, Ju Wang, Lin Xiao, Yubing Wang, Maogang Shen, Jiayi Liao, Yifei Zheng, Joris Hemelaar

**Affiliations:** ^1^Nuffield Department of Population Health, Infectious Disease Epidemiology Unit, University of Oxford, Oxford, United Kingdom; ^2^School of Public Health, Chengdu University of Traditional Chinese Medicine, Chengdu, Sichuan, China; ^3^Liangshan Prefecture Centre for Disease Control and Prevention, Xichang, Sichuan, China; ^4^Sichuan Provincial Center for Disease Control and Prevention, Center for AIDS/STD Control and Prevention, Chengdu, Sichuan, China

**Keywords:** HIV-1, treatment failure, drug resistance, subtype, antiretroviral therapy, mutation

## Abstract

**Introduction:**

Despite expanded antiretroviral therapy (ART) in China, HIV transmission persists. Liangshan Prefecture is one of the areas in China most severely affected by HIV, with high levels of drug resistance. A deeper understanding of HIV-1 drug resistance can lead to improvements in current treatment policies.

**Methods:**

We conducted an analysis of HIV drug resistance mutations (DRMs) among patients with treatment failure and people newly diagnosed with HIV in Liangshan Prefecture. 8,523 blood samples were collected from people living with HIV with treatment failure and newly diagnosed individuals in all 15 counties and two cities in Liangshan Prefecture between 2021 and 2023.

**Results:**

43.0% of patients with treatment failure acquired HIV through the heterosexual route, followed by injecting drug use (38.7%), while newly diagnosed individuals mainly acquired HIV through the heterosexual route (86.7%). 95.6% of patients with treatment failure were infected with HIV-1 variant CRF07_BC and 2.7% with CRF08_BC, and newly diagnosed individuals were also main infected with HIV-1 variant CRF07_BC (90.9), followed by CRF08_BC (4.0%) and CRF01_AE (2.5%). The overall prevalence of acquired drug resistance (ADR) among patients with treatment failure was 57.4%. The overall prevalence of pre-treatment drug resistance (PDR) among newly diagnosed individuals was 23.9%. A high prevalence of ADR and PDR (especially high-level resistance) to efavirenz (48.0% vs. 11.1%) and nevirapine (49.6% vs. 11.4%) was found. The main non-nucleoside reverse transcriptase inhibitor (NNRTI)-associated ADR and PDR mutations were K103, V106, and V179. Our findings highlight age <18 years, injecting drug use, and initiation on NNRTI-based regimen as independent risk factors for HIV ADR development. We found minor variants as a risk factor for PDR, and CRF01_AE was associated with a higher risk than CRF07_BC for nucleoside reverse transcriptase inhibitor (NRTI) PDR.

**Discussion:**

Given the high levels of NNRTI ADR and PDR, future clinical treatment plans should minimize the use of NNRTI-based regimens and should instead adopt alternative ART regimens more frequently.

## 1 Introduction

HIV/AIDS remains a major health problem worldwide. According to the Joint United Nations Program on HIV/AIDS (UNAIDS), approximately 39.9 million people globally were living with HIV, and 30.7 million of them were accessing antiretroviral therapy (ART) in 2023 (1). In China, HIV/AIDS is a significant health issue, with around 1,280,700 people living with HIV (PLWH) at the end of December 2023 (2). The HIV epidemic in China is unevenly distributed, and the province of Sichuan has the highest number of PLWH (3, 4).

Treatment options for HIV/AIDS have expanded significantly in recent years, with ART proving highly effective at reducing disease progression and improving the quality of life for PLWH (5–7). However, drug resistance has been a key challenge in the management and control of HIV-1, impacting the success of antiretroviral treatment, and heightening HIV-related morbidity and mortality (8, 9). Drug resistance mutations (DRMs) are the major cause of antiretroviral treatment failure (10). Since the initiation of the National Free Antiretroviral Treatment Program (NFATP) by the Chinese government in 2003, drug resistance has risen with the escalation of treatment coverage (11). Despite expanded ART access in China, drug resistance could, therefore, threaten ART effectiveness. DRMs to nucleoside reverse transcriptase inhibitors (NRTIs) and non-nucleoside reverse transcriptase inhibitors (NNRTIs) are complex and common and were the most common cause of first-line antiretroviral treatment failure (12, 13).

Drug resistance can be divided into primary drug resistance and acquired drug resistance (ADR). Primary drug resistance can be divided into pre-treatment drug resistance (PDR) and transmitted drug resistance (TDR) (14). TDR refers to HIV drug resistance present when a new HIV infection is acquired, whether through horizontal or vertical transmission; PDR is a broader term encompassing TDR and any HIV drug resistance present at the initiation of ART and is more commonly used to describe infections of unknown duration and when TDR cannot be conclusively ascertained (15). PDR can result in the failure of first-line regimens, especially when not recognized at the time of ART initiation (16). The prevalence of PDR and ADR in China varies significantly across regions (17), with the reported PDR ranging from 5.5% in Sichuan (3) to 18.3% in Xian (18), and ADR ranging from approximately 46.6% in Chongqing (19) to 81.2% in Henan (20).

Liangshan Prefecture, an autonomous prefecture with the largest population of Yi people in Sichuan Province, is located on the key route through which drugs from the “Golden Triangle” flow into mainland China (21). Liangshan Prefecture is one of the areas most severely affected by HIV/AIDS in China and had a total of 41,623 PLWH in 2019 (22). Within Liangshan Prefecture, prevalence rates of HIV are over 1% in Butuo County, Zhaojue County, Meigu county, Yuexi County, and Jinyang County, which are the counties with the highest HIV prevalence in China (23). In Liangshan Prefecture, CRF07_BC was the main HIV variant of drug users in the past two decades (24). While the HIV epidemic in Liangshan was initially driven by injecting drug use, sexual transmission became the predominant mode of HIV transmission in Liangshan Prefecture from 2014 (25). The genotype of HIV-1 is related to the route of transmission, and there are differences in epidemic scale and distribution characteristics of HIV-1 variants (26, 27). Liangshan Prefecture is an important labor-exporting area, with socioeconomic development and the growth of population mobility. HIV-1 infected populations moving frequently may result in a more wide-spread and complex distribution of HIV-1 genotypes, which may increase the risk of virological failure of antiretroviral treatment, increase the spread of drug-resistant strains, and bring a huge challenge to controlling the HIV epidemic (28). To date, there has not been any large-scale study on HIV drug resistance in Liangshan Prefecture. We conducted an analysis of HIV DRMs among patients with treatment failure and newly diagnosed people in Liangshan, aiming to delineate the characteristics of DRM and their associated risk factors.

## 2 Materials and methods

### 2.1 Study population and sample collection

**Figure 1:**
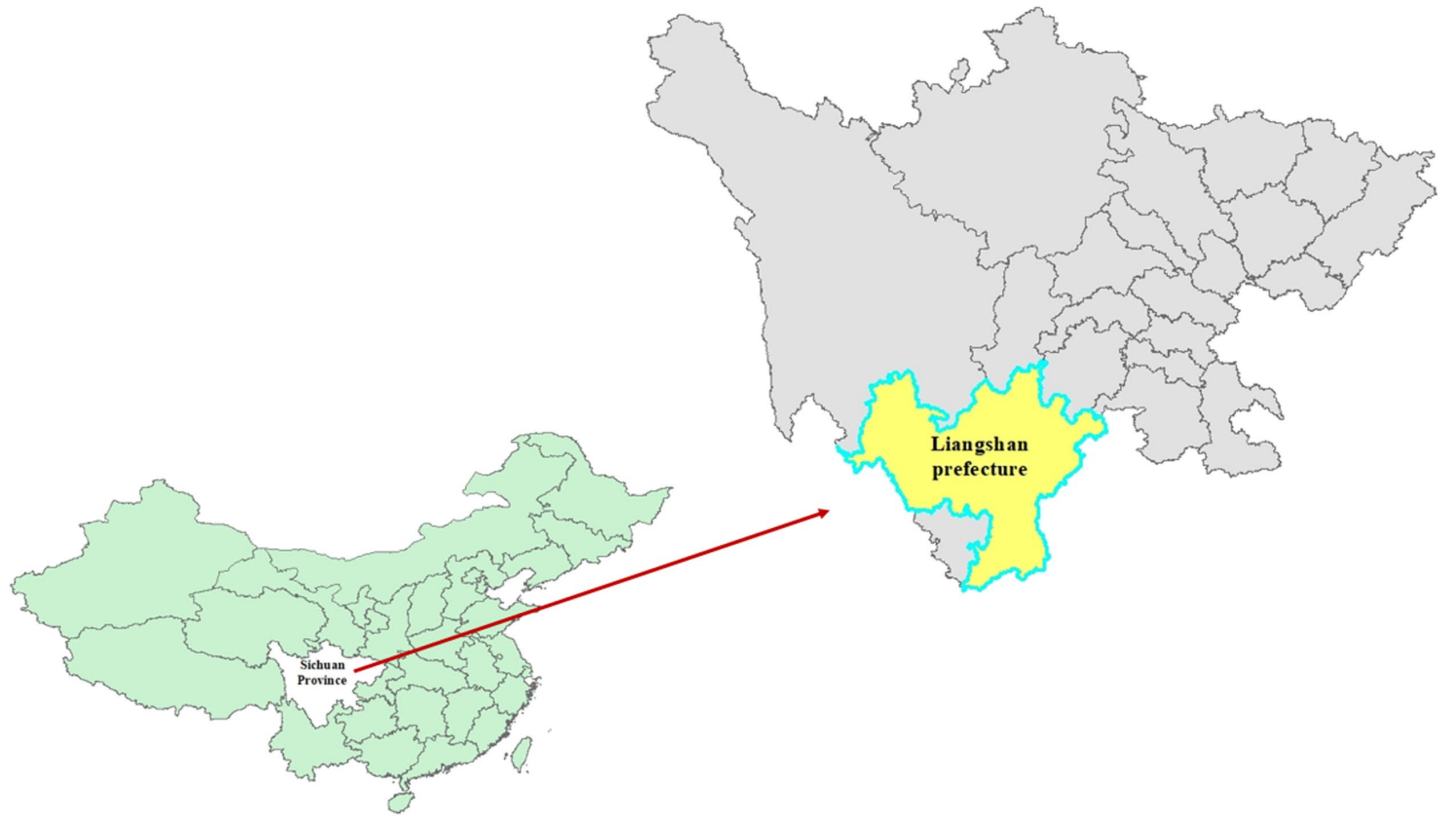
The geographical location of Liangshan Prefecture within Sichuan Province in China.

Liangshan Prefecture is located in the southwestern mountainous area of Sichuan Province (Figure 1). The area primarily depends on agriculture and transportation infrastructure is basic, resulting in a relatively low level of economic development. Blood samples were collected for testing from all 15 counties and 2 cities in Liangshan Prefecture between 2021 and 2023. The samples included all patients with treatment failure (defined as having viral load ≥1,000 copies/ml or being assessed by physicians as having poor treatment outcomes) reported to Center for Disease Control and Prevention (CDC) as well as newly diagnosed individuals.

Plasma samples were collected in accordance with standard procedures (29) by laboratory personnel of the local CDC and were transported to the Sichuan Provincial Center for Disease Control and Prevention for HIV-1 drug resistance testing (30).

Patients’ demographic information—including sex, age, marital status, ethnicity, education level, and transmission route—was collected from the National HIV/AIDS Comprehensive Response Information Management System, a web-based real-time database managed by the National Center for AIDS/STD Control and Prevention (NCAIDS) of the Chinese Center for Disease Control and Prevention (CDC) (31).

### 2.2 HIV-1 gene amplification and drug resistance analysis

Viral nucleic acid was obtained from 200 μL plasma of PLWH by extraction machines (MagNA Pure LC system, Roche, Branchburg, NJ). HIV-1 *pol* sequences were amplified and sequenced. Reverse Transcription-Polymerase Chain Reaction (RT-PCR) was used to amplify the full-length protease gene and the first 300 codons of the reverse transcriptase gene. Two rounds of PCR amplification were used, following the HIV-1 Genotype Drug Resistance Detection and Quality Assurance Guidelines (2013 Edition) (3, 32). The target bands were subjected to 1% agarose gel electrophoresis for validation, and the amplified product was sequenced. Sequences were then spliced and edited, and were submitted to the drug resistance database of Stanford University^1^ for resistance mutation analysis.

For HIV-1 subtyping, the HIV sequences were aligned with HIV-1 reference sequences available in the Los Alamos National Laboratory database.^2^ Multiple alignments were made automatically using Mega version 7.0 with minor manual adjustments.

Each drug resistance mutation is assigned a drug penalty score. The total score for a drug is derived by adding the scores for all mutations associated with resistance to that drug to infer 1 of 5 levels of resistance: susceptible, potential low-level resistance, low-level resistance, intermediate resistance, and high-level resistance (33–35).

### 2.3 Statistical analysis

Demographic characteristics of the study participants were summarized using frequencies and percentages. Multivariate logistic regression was employed to assess the risk factors for HIV PDR and ADR. Unadjusted odds ratios (OR), adjusted odds ratios (AOR), and their respective 95% confidence intervals (CI) were calculated. Significance was established at *p* < 0.05. IBM SPSS 22 software was utilized for statistical analysis.

## 3 Results

### 3.1 Participant characteristics

**Figure 2:**
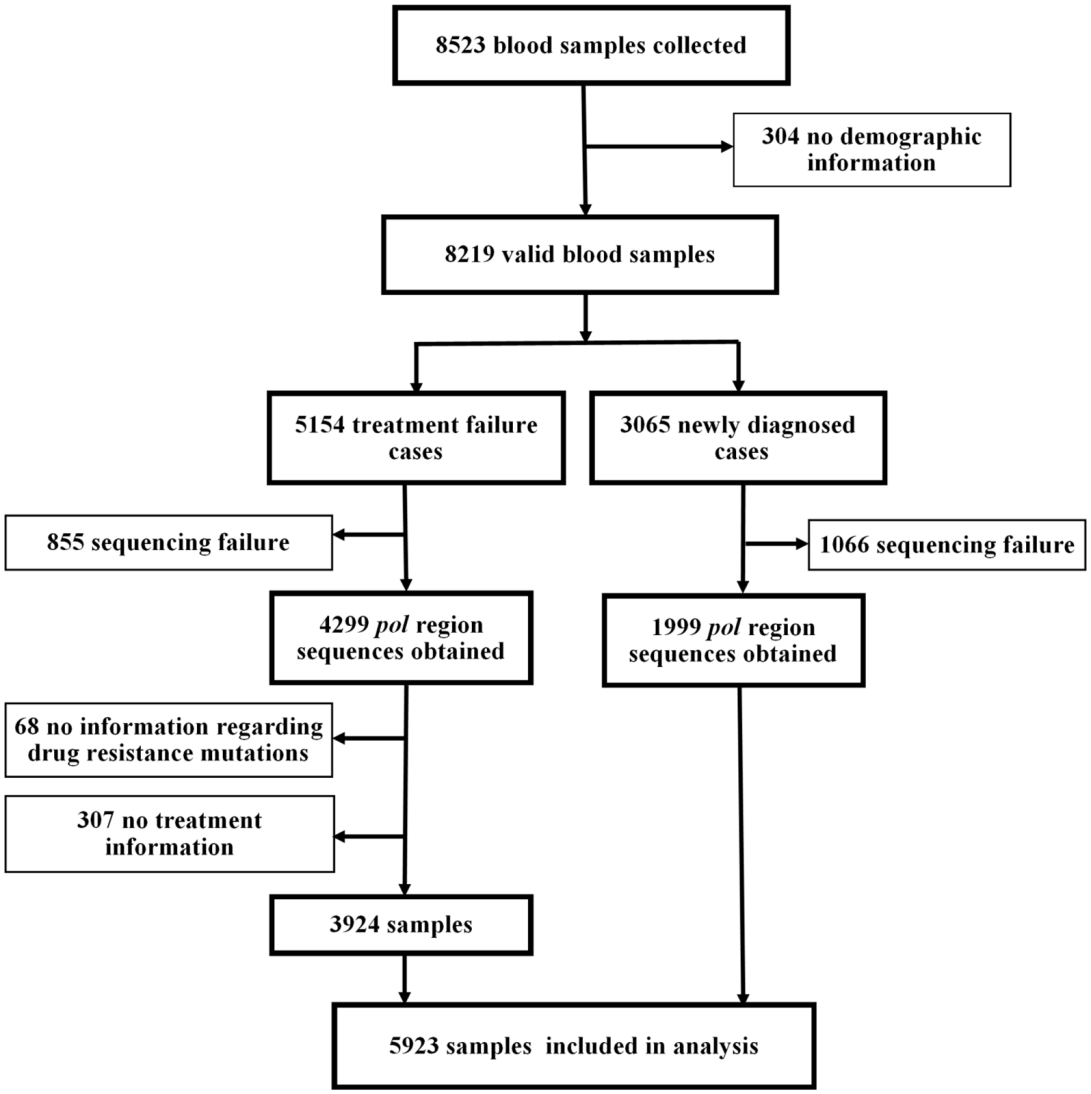
Study profile.

A total of 8,523 participants were enrolled in this study (Figure 2). After excluding samples with sequencing failure and missing information, the final analysis included 3,924 individuals with treatment failure for ADR analysis and 1,999 newly diagnosed individuals for PDR analysis.

Characteristics of patients with treatment failure are summarized in Table 1. Of the 3,924 individuals with treatment failure in this study, the majority were male (71.5%). 63.8% were aged 25–44 years and 92.3% were of Yi ethnicity. The most common mode of HIV-1 transmission was heterosexual contact (43.0%), followed by injecting drug use (38.7%). Regarding education, 57.2% were illiterate, and 33.6% attended primary schooling. The primary occupation was farming, comprising 75.1%. The most prevalent variant of HIV-1 was CRF07_BC (95.6%), followed by CRF08_BC (2.7%) and CRF01_AE (0.8%). ART regimens mainly consisted of TDF + 3TC + EFV (73.4%), AZT + 3TC+ NVP (11.0%) and AZT + 3TC+ EFV (9.3%).

**Table 1:** Characteristics of HIV patients with treatment failure in Liangshan Prefecture, China.

Variable	Total (*n* = 3,924)	No drug resistance (*n* = 1,670)	Drug resistance			
			PI (*n* = 123)	NRTI (*n* = 934)	NNRTI (*n* = 2,156)	ADR (*n* = 2,254)
Time since diagnosis (years)						
1–5	1865 (47.5)	770 (46.1)	51 (41.5)	458 (49.0)	1,049 (48.7)	1,095 (48.6)
6–10	1,136 (29.0)	484 (29.0)	35 (28.5)	261 (27.9)	622 (28.8)	652 (28.9)
>10	923 (23.5)	416 (24.9)	37 (30.1)	215 (23.0)	485 (22.5)	507 (22.5)
Gender						
Male	2,806 (71.5)	1,179 (70.6)	97 (78.9)	619 (66.3)	1,565 (72.6)	1,627 (72.2)
Female	1,118 (28.5)	491 (29.4)	26 (21.1)	315 (33.7)	591 (27.4)	627 (27.8)
Age (years)						
<18	453 (11.5)	113 (6.8)	24 (19.5)	237 (25.4)	327 (15.2)	340 (15.1)
18–24	152 (3.9)	75 (4.5)	5 (4.1)	28 (3.0)	76 (3.5)	77 (3.4)
25–44	2,505 (63.8)	1,119 (67.0)	72 (58.5)	481 (51.5)	1,325 (61.5)	1,386 (61.5)
45–64	709 (18.1)	304 (18.2)	21 (17.1)	173 (18.5)	384 (17.8)	405 (18.0)
≥65	105 (2.7)	59 (3.5)	1 (0.8)	15 (1.6)	44 (2.0)	46 (2.0)
Ethnicity						
Yi	3,621 (92.3)	1,529 (91.6)	115 (93.5)	869 (93.0)	2004 (92.9)	2092 (92.8)
Han	288 (7.3)	135 (8.1)	8 (6.5)	61 (6.5)	143 (6.6)	153 (6.8)
Others	15 (0.4)	6 (0.4)	0	4 (0.4)	9 (0.4)	9 (0.4)
Transmission route						
Heterosexual	1,688 (43.0)	817 (48.9)	42 (34.1)	375 (40.1)	825 (38.3)	871 (38.6)
Injecting drug use	1,518 (38.7)	626 (37.5)	46 (37.4)	261 (27.9)	857 (39.7)	892 (39.6)
Mother to child transmission	352 (9.0)	88 (5.3)	22 (17.9)	190 (20.3)	255 (11.8)	264 (11.7)
Men who have sex with men	17 (0.4)	6 (0.4)	0	4 (0.4)	11 (0.5)	11 (0.5)
Heterosexual +Injecting drug use	190 (4.8)	86 (5.1)	7 (5.7)	37 (4.0)	101 (4.7)	104 (4.6)
Uncertain	159 (4.1)	47 (2.8)	6 (4.9)	67 (7.2)	107 (5.0)	112 (5.0)
Education						
Illiterate	2,246 (57.2)	946 (56.6)	77 (62.6)	568 (60.8)	1,239 (57.5)	1,300 (57.7)
Primary school	1,320 (33.6)	568 (34.0)	37 (30.1)	286 (30.6)	721 (33.4)	752 (33.4)
Junior high school	278 (7.1)	116 (6.9)	7 (5.7)	60 (6.4)	157 (7.3)	162 (7.2)
High school or technical secondary school	65 (1.7)	32 (1.9)	1 (0.8)	16 (1.7)	33 (1.5)	33 (1.5)
College or above	15 (0.4)	8 (0.5)	1 (0.8)	4 (0.4)	6 (0.3)	7 (0.3)
Marital status						
Married or cohabiting	2060 (52.5)	933 (55.9)	59 (48.0)	434 (46.5)	1,077 (50.0)	1,127 (50.0)
Unmarried	1,347 (34.3)	513 (30.7)	47 (38.2)	413 (44.2)	802 (37.2)	834 (37.0)
Divorced or widowed	461 (11.7)	205 (12.3)	13 (10.6)	73 (7.8)	243 (11.3)	256 (11.4)
Unknown	56 (1.4)	19 (1.1)	4 (3.3)	14 (1.5)	34 (1.6)	37 (1.6)
Occupation						
Farmer	2,947 (75.1)	1,316 (78.8)	83 (67.5)	605 (64.8)	1,553 (72.0)	1,631 (72.4)
Laborer	69 (1.8)	33 (2.0)	3 (2.4)	16 (1.7)	34 (1.6)	36 (1.6)
Unemployed	197 (5.0)	86 (5.1)	5 (4.1)	29 (3.1)	110 (5.1)	111 (4.9)
Government and public institutions	7 (0.2)	3 (0.2)	0	2 (0.2)	4 (0.2)	4 (0.2)
Business services	11 (0.3)	5 (0.3)	0	1 (0.1)	5 (0.2)	6 (0.3)
Children and students	481 (12.3)	122 (7.3)	26 (21.1)	248 (26.6)	346 (16.0)	359 (15.9)
Others	212 (5.4)	105 (6.3)	6 (4.9)	33 (3.5)	104 (4.8)	107 (4.7)
HIV-1 variants						
CRF01_AE	33 (0.8)	14 (0.8)	0	9 (1.0)	19 (0.9)	19 (0.8)
CRF07_BC	3,752 (95.6)	1,596 (95.6)	119 (96.7)	897 (96.0)	2061 (95.6)	2,156 (95.7)
CRF08_BC	104 (2.7)	46 (2.8)	4 (3.3)	21 (2.2)	55 (2.6)	58 (2.6)
URFs	25 (0.6)	20 (0.7)	0	4 (0.4)	13 (0.6)	13 (0.6)
Minor	10 (0.3)	2 (0.2)	0	3 (0.3)	8 (0.4)	8 (0.3)
Recent CD4+ T lymphocyte count (pcs/μL)						
<200	717 (18.3)	295 (17.7)	25 (20.3)	178 (19.1)	403 (18.7)	422 (18.7)
200–350	1,134 (28.9)	475 (28.4)	37 (30.1)	270 (28.9)	626 (29.0)	659 (29.2)
>350	2065 (52.6)	896 (53.7)	61 (49.6)	485 (51.9)	1,123 (52.1)	1,169 (51.9)
Missing	8 (0.2)	4 (0.2)	0	1 (0.1)	4 (0.2)	4 (0.2)
ART regimen at treatment failure						
**NRTI + NNRTI**	**3,767 (96.0)**	**1,584 (94.9)**	**119 (96.7)**	**909 (97.3)**	**2096 (97.2)**	**2,183 (96.9)**
TDF + 3TC + EFV	2,879 (73.4)	1,286 (77.0)	77 (62.6)	550 (58.9)	1,523 (70.6)	1,593 (70.7)
TDF + 3TC + NVP	29 (0.7)	8 (0.5)	0	9 (1.0)	21 (1.0)	21 (0.9)
TDF + NVP + EFV	2 (0.1)	1 (0.1)	0	1 (0.1)	1 (0.05)	1 (0.04)
TDF + D4T + EFV	1 (0.03)	0	0	0	1 (0.05)	1 (0.04)
AZT + 3TC + EFV	363 (9.3)	139 (8.3)	16 (13.0)	115 (12.3)	219 (10.2)	224 (9.9)
AZT + 3TC + NVP	433 (11.0)	124 (7.4)	23 (18.7)	213 (22.8)	298 (13.8)	309 (13.7)
ABC + 3TC + EFV	5 (0.1)	0	0	3 (0.3)	5 (0.2)	5 (0.2)
ABC + 3TC + NVP	5 (0.1)	2 (0.1)	0	0	3 (0.1)	3 (0.1)
D4T + 3TC + EFV	5 (0.1)	2 (0.1)	0	2 (0.2)	3 (0.1)	3 (0.1)
D4T + 3TC + NVP	43 (1.1)	21 (1.3)	3 (2.4)	0	21 (1.0)	22 (1.0)
DDI + 3TC + EFV	1 (0.03)	0	0	2 (0.2)	1 (0.05)	1 (0.04)
3TC+ EFV	1 (0.03)	1 (0.1)	0	0	0	0
**NRTI + PI**	**152 (3.9)**	**84 (5.0)**	**4 (3.3)**	**23 (2.5)**	**57 (2.6)**	**68 (3.0)**
TDF + 3TC + LPV/r	59 (1.5)	30 (1.8)	1 (0.8)	6 (0.6)	25 (1.2)	29 (1.3)
AZT + 3TC + LPV/r	86 (2.2)	50 (3.0)	3 (2.4)	0	29 (1.3)	36 (1.6)
3TC + LPV/r	7 (0.2)	4 (0.2)	0	0	3 (0.1)	3 (0.1)
**Triple NRTI**	**4 (0.1)**	**1 (0.1)**	**0**	**2 (0.2)**	**3 (0.1)**	**3 (0.1)**
TDF + 3TC + AZT	3 (0.1)	1 (0.1)		1 (0.1)	2 (0.1)	2 (0.1)
ABC + 3TC+ AZT	1 (0.03)	0		1 (0.1)	1 (0.05)	1 (0.04)
**PI monotherapy**	**1 (0.03)**	**1 (0.1)**	**0**	**0**	**0**	**0**
LPV/r	1 (0.03)	1 (0.1)				

*Minor: Subtype A (1), Subtype B (3), CRF55_01B (1), CRF77_cpx (1), CRF85_BC (1), CRF88_BC (1), CRF105_0108 (2). ABC, Abacavir; ADR, Acquired drug resistance; AZT, Azidothymidine; D4T, Stavudine; DDI, Didanosine; EFV, Efavirenz; LPV/r, lopinavir/ritonavir; NNRTI, Non-Nucleoside Reverse Transcriptase Inhibitor; NVP, nevirapine; NRTI, Nucleoside Reverse Transcriptase Inhibitor; PI, Protease inhibitor; 3TC, Lamivudine; TDF, Tenofovir disoproxil fumarate; URFs, Unique Recombinant Forms. ART classes are displayed in bold.

Among the 1,999 newly diagnosed individuals in the study, 53.5% were male, and 50.6% were aged 25–44 years (Table 2). The majority of newly diagnosed individuals acquired HIV through heterosexual contact (86.7%). Fewer newly diagnosed patients were illiterate (45.9%) and more completed primary schooling (38.8%), compared to treatment failure patients. Most newly diagnosed patients were farmers (83.7%). Compared to treatment failure patients, the HIV-1 variants among newly diagnosed patients were more diverse, with a lower proportion of CRF07_BC (90.9%), and a higher prevalence of CRF08_BC (4.0%) and CRF01_AE (2.5%).

**Table 2:** Characteristics of people newly diagnosed with HIV in Liangshan Prefecture, China.

Variable	Total (*n* = 1999)	No drug resistance (*n* = 1,521)	Drug resistance			
			PI (*n* = 55)	NRTI (*n* = 72)	NNRTI (*n* = 409)	PDR (*n* = 478)
Gender						
Male	1,070 (53.5)	823 (54.1)	33 (60.0)	39 (54.2)	210 (51.3)	247 (51.7)
Female	929 (46.5)	698 (45.9)	22 (40.0)	33 (45.8)	199 (48.7)	231 (48.3)
Age (years)						
<18	207 (10.4)	142 (9.3)	10 (18.2)	10 (13.9)	56 (13.7)	65 (13.6)
18–24	178 (8.9)	139 (9.1)	4 (7.3)	10 (13.9)	30 (7.3)	39 (8.2)
25–44	1,011 (50.6)	763 (50.2)	30 (54.5)	30 (41.7)	216 (52.8)	248 (51.9)
45–64	501 (25.1)	394 (25.9)	10 (18.2)	21 (29.2)	89 (21.8)	107 (22.4)
≥65	102 (5.1)	83 (5.5)	1 (1.8)	1 (1.4)	18 (4.4)	19 (4.0)
Ethnicity						
Yi	1705 (85.3)	1,294 (85.1)	45 (81.8)	60 (83.3)	354 (86.6)	411 (86.0)
Han	271 (13.6)	211 (13.9)	10 (18.2)	9 (12.5)	49 (12.0)	60 (12.6)
Others	23 (1.2)	16 (1.1)	0	3 (4.2)	6 (1.5)	7 (1.5)
Transmission route						
Heterosexual	1734 (86.7)	1,332 (87.6)	44 (80.0)	61 (84.7)	343 (83.9)	402 (84.1)
Injecting drug use	37 (1.9)	25 (1.6)	1 (1.8)	1 (1.4)	11 (2.7)	12 (2.5)
Mother to child transmission	83 (4.2)	58 (3.8)	1 (1.8)	5 (6.9)	23 (5.6)	25 (5.2)
Men who have sex with men	13 (0.7)	9 (0.6)	1 (1.8)	0	3 (0.7)	4 (0.8)
Heterosexual +Injecting drug use	12 (0.6)	11 (0.7)	0	0	1 (0.2)	1 (0.2)
Uncertain	120 (6.0)	86 (5.7)	8 (14.5)	5 (6.9)	28 (6.8)	34 (7.1)
Education						
Illiterate	917 (45.9)	687 (45.2)	21 (38.2)	29 (40.3)	207 (50.6)	230 (48.1)
Primary school	776 (38.8)	603 (39.6)	21 (38.2)	26 (36.1)	144 (35.2)	173 (36.2)
Junior high school	204 (10.2)	153 (10.1)	8 (14.5)	8 (11.1)	43 (10.5)	51 (10.7)
High school or technical secondary school	52 (2.6)	38 (2.5)	2 (3.6)	6 (8.3)	9 (2.2)	14 (2.9)
College or above	50 (2.5)	40 (2.6)	3 (5.5)	3 (4.2)	6 (1.5)	10 (2.1)
Marital status						
Married or cohabiting	870 (43.5)	689 (45.3)	21 (38.2)	22 (30.6)	159 (38.9)	181 (37.9)
Unmarried	568 (28.4)	411 (27.0)	20 (36.4)	30 (41.7)	129 (31.5)	157 (32.8)
Divorced or widowed	556 (27.8)	418 (27.5)	14 (25.5)	20 (27.8)	119 (29.1)	138 (28.9)
Unknown	5 (0.3)	3 (0.2)	0	0	2 (0.5)	2 (0.4)
Occupation						
Farmer	1,674 (83.7)	1,285 (84.5)	40 (72.7)	57 (79.2)	338 (82.6)	389 (81.4)
Laborer	25 (1.3)	21 (1.4)	0	0	4 (1.0)	4 (0.8)
Unemployed	36 (1.8)	24 (1.6)	3 (5.5)	4 (5.6)	6 (1.5)	12 (2.5)
Government and public institutions	31 (1.6)	24 (1.6)	0	2 (2.8)	6 (1.5)	7 (1.5)
Business services	10 (0.5)	9 (0.6)	0	0	1 (0.2)	1 (0.2)
Children and students	189 (9.5)	131 (8.6)	10 (18.2)	9 (12.5)	49 (12.0)	58 (12.1)
Others	34 (1.7)	27 (1.8)	2 (3.6)	0	5 (1.2)	7 (1.5)
HIV-1 variants						
CRF01_AE	50 (2.5)	35 (2.3)	3 (5.5)	7 (9.7)	9 (2.2)	15 (3.1)
CRF07_BC	1817 (90.9)	1,392 (91.5)	49 (89.1)	58 (80.6)	366 (89.5)	425 (88.9)
CRF08_BC	80 (4.0)	63 (4.1)	1 (1.8)	4 (5.6)	13 (3.2)	17 (3.6)
URFs	31 (1.6)	21 (1.4)	2 (3.6)	3 (4.2)	10 (2.4)	10 (2.1)
Minor	21 (1.1)	10 (0.7)	0	0	11 (2.7)	11 (2.3)
Recent CD4+ T lymphocyte count (pcs/μL)						
<200	218 (10.9)	148 (9.7)	9 (16.4)	12 (16.7)	58 (14.2)	70 (14.6)
200–350	511 (25.6)	377 (24.8)	15 (27.3)	23 (31.9)	113 (27.6)	134 (28.0)
>350	1,162 (58.1)	912 (60.0)	27 (49.1)	34 (47.2)	217 (53.1)	250 (52.3)
Missing	108 (5.4)	84 (5.5)	4 (7.3)	3 (4.2)	21 (5.1)	24 (5.0)

*Minor: Subtype B (2), Subtype C (2), CRF55_01B (5), CRF 67_01B (1), CRF83_cpx (3), CRF85_BC (1), CRF88_BC (2), CRF105_0108 (5). NNRTI, Non-Nucleoside Reverse Transcriptase Inhibitor; NRTI, Nucleoside Reverse Transcriptase Inhibitor; PDR, Pre-treatment drug resistance; PI, Protease inhibitor; URFs, Unique Recombinant Forms.

### 3.2 ADR and PDR prevalence

**Figure 3:**
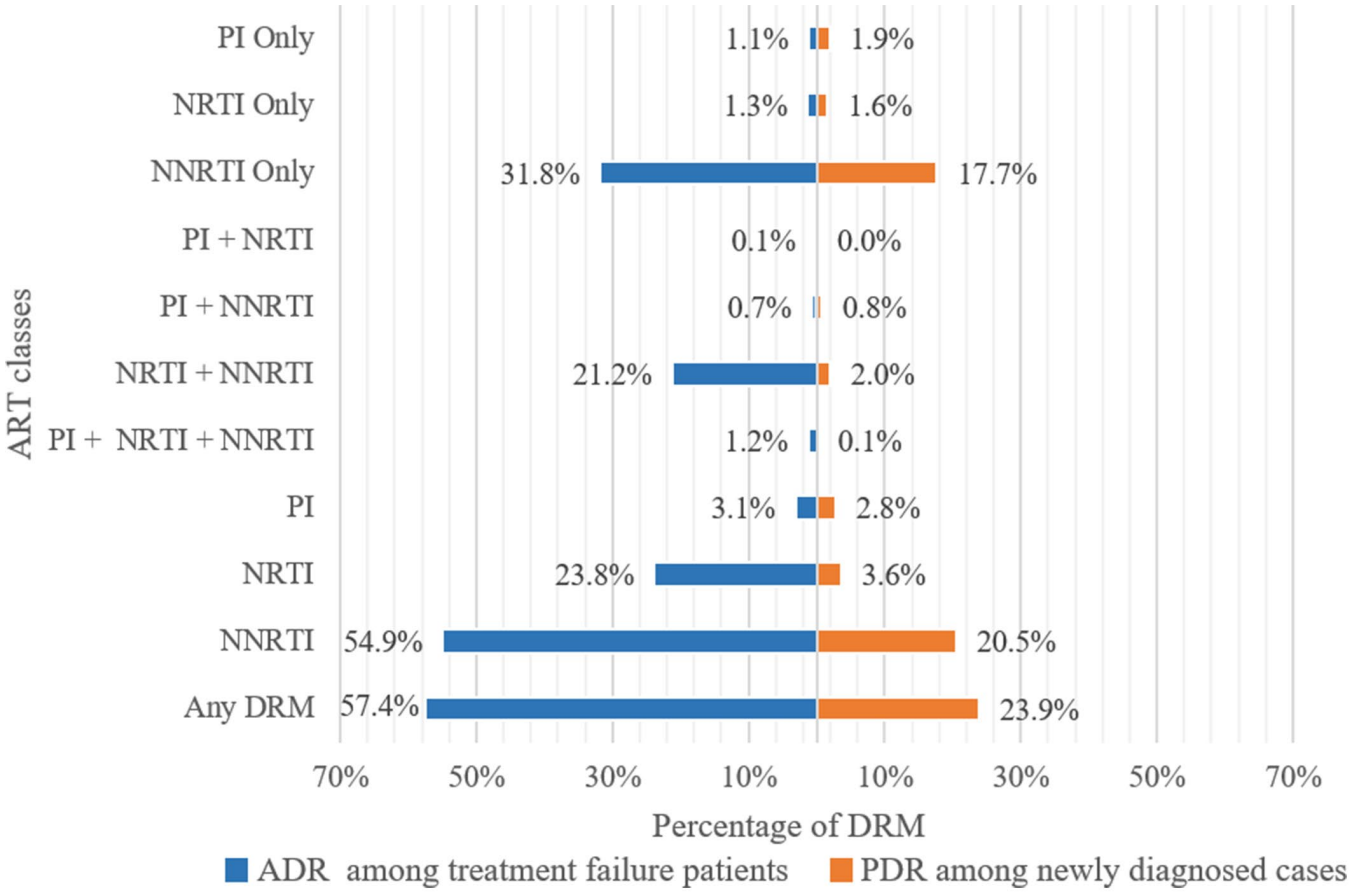
Frequency of drug resistance to ART classes. ADR, Acquired drug resistance; PDR, Pre-treatment drug resistance; PI, Protease inhibitor; NRTI, Nucleoside Reverse Transcriptase Inhibitor; NNRTI, Non-Nucleoside Reverse Transcriptase Inhibitor; DRM, Drug Resistance Mutation.

Of 3,924 *pol* sequences from individuals with treatment failure, 57.4% had one or more resistance mutations (Figure 3). The prevalence of ADR to NNRTI was 54.9%, followed by ADR to NRTI at 23.8%, and ADR to PI at 3.1%. Specifically, resistance to only NNRTI, NRTI, and PI were 31.8, 1.3, and 1.1%, respectively. The prevalence of dual-class resistance to NNRTI+NRTI was 21.2%, and the occurrence of triple-drug resistance (NNRTI+NRTI+PI) was relatively low at 1.2% (Figure 3).

The prevalence of PDR among 1999 newly diagnosed individuals was 23.9%. The prevalence of PDR to NNRTI was 20.5%, followed by PDR to NRTI at 3.6%, and PDR to PI at 2.8% (Figure 3). Resistance to only NNRTI, NRTI, and PI were 17.7, 1.6, and 1.9%, respectively. The prevalence of PDR to only NNRTI (17.7%) was lower than of ADR, while the prevalence of PDR to only NRTI (1.6%) and PI (1.9%) was higher than of ADR. The most prevalent dual-class resistance was against NNRTI+NRTI, accounting for 2.0% of newly diagnosed individuals. The occurrence of triple-class (NNRTI+NRTI+PI) PDR was low at 0.1% (Figure 3).

### 3.3 Resistance to individual antiviral drugs

**Figure 4:**
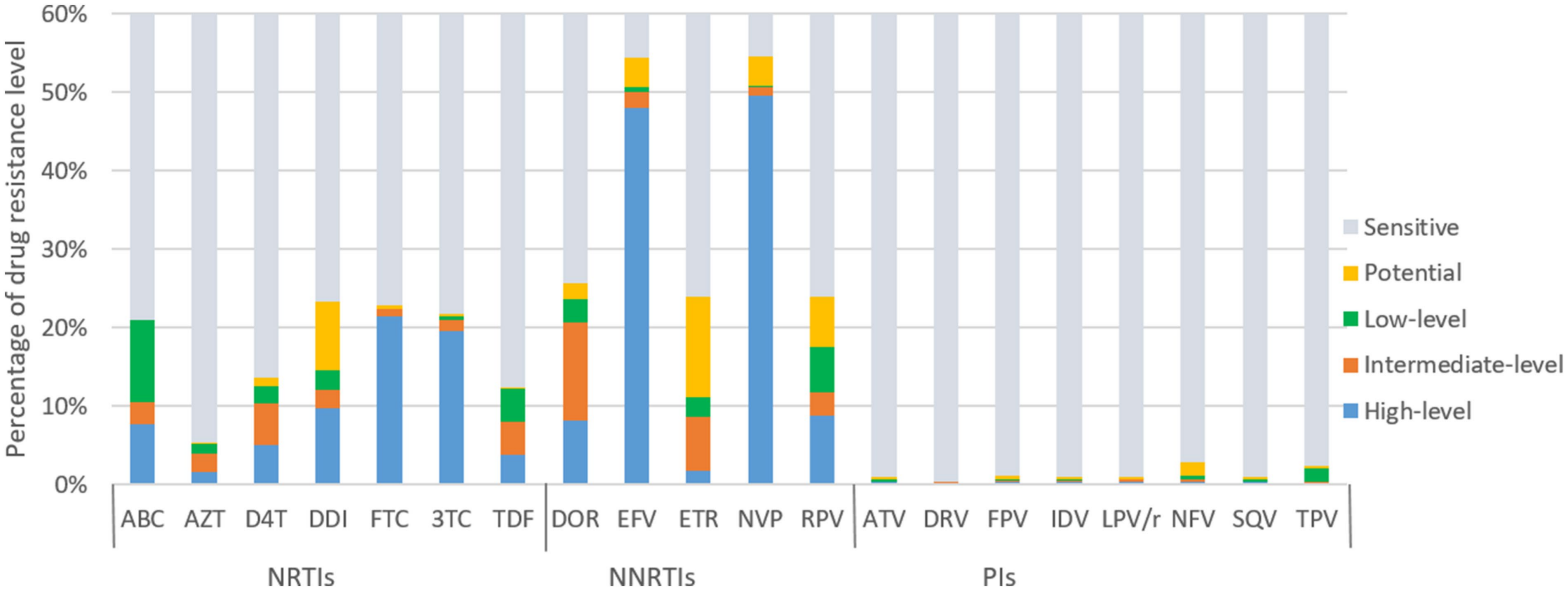
ART drug resistance among HIV patients with treatment failure in Liangshan Prefecture, China. ABC, Abacavir; AZT, Azidothymidine; D4T, Stavudine; DDI, Didanosine; FTC, Emtricitabine; 3TC, Lamivudine; TDF, Tenofovir disoproxil fumarate; DOR, Doravirine; EFV, Efavirenz; ETR, etravirine; NVP, Nevirapine; RPV, Rilpivirine; ATV, Atazanavir; DRV, Darunavir; FPV, Fosamprenavir; IDV, Indinavir; LPV/r, lopinavir/ritonavir; NFV, Nelfinavir; SQV, Saquinavir; TPV, Tipranavir; NRTI, Nucleoside Reverse Transcriptase Inhibitor; NNRTI, Non-Nucleoside Reverse Transcriptase Inhibitor; PI, Protease inhibitor.

Figure 4 illustrates the predicted resistance of 3,924 individuals with treatment failure from Liangshan to 20 antiretroviral drugs. For NRTIs, the virus exhibited high/intermediate level resistance to FTC, 3TC, DDI, ABC, D4T, TDF and AZT at frequencies of 22.4, 21.0, 12.1, 10.5, 10.4, 8.0 and 4.0%, respectively. For NNRTIs, NVP, EFV, DOR, RPV and ETR had high/intermediate level frequency of 50.6, 50.1, 20.7, 11.8 and 8.6%, respectively. NVP (54.6%) and EFV (54.5%) were the most common NNRTI drugs with ADR. Conversely, frequencies of high/intermediate level to PI drugs remained low, all below 1%.

**Figure 5:**
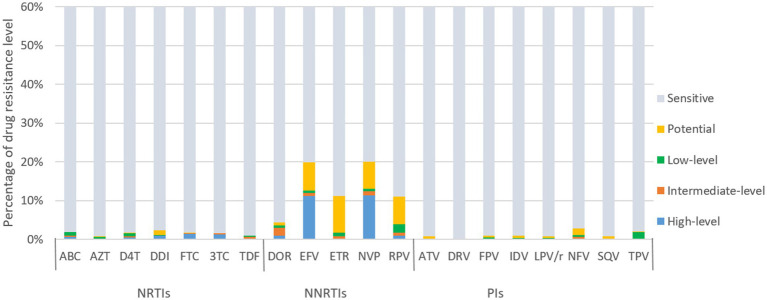
ART drug resistance among people newly diagnosed with HIV in Liangshan Prefecture, China. ABC, Abacavir; AZT, Azidothymidine; D4T, Stavudine; DDI, Didanosine; FTC, Emtricitabine; 3TC, Lamivudine; TDF, Tenofovir disoproxil fumarate; DOR, Doravirine; EFV, Efavirenz; ETR, etravirine; NVP, Nevirapine; RPV, Rilpivirine; ATV, Atazanavir; DRV, Darunavir; FPV, Fosamprenavir; IDV, Indinavir; LPV/r, lopinavir/ritonavir; NFV, Nelfinavir; SQV, Saquinavir; TPV, Tipranavir; NRTI, Nucleoside Reverse Transcriptase Inhibitor; NNRTI, Non-Nucleoside Reverse Transcriptase Inhibitor; PI, Protease inhibitor.

In contrast, newly diagnosed individuals showed lower resistance frequencies to 20 antiretroviral drugs than treatment failure patients (Figure 5). For NRTIs, the virus exhibited high/intermediate level resistance to FTC, 3TC, DDI, ABC, AZT, D4T and TDF at frequency of 1.6, 1.5, 1.0, 0.9, 0.8 and 0.6%, respectively. For NNRTIs, NVP, EFV, DOR, RPV and ETR had high/intermediate level frequency of 12.5, 11.9, 2.9, 1.7 and 0.8%, respectively. NVP (20.1%) and EFV (19.9%) were the most common NNRTI drugs with PDR. The frequencies of high/intermediate levels among newly diagnosed individuals to PIs drugs were all below 1%.

### 3.4 Drug resistance mutations

**Figure 6:**
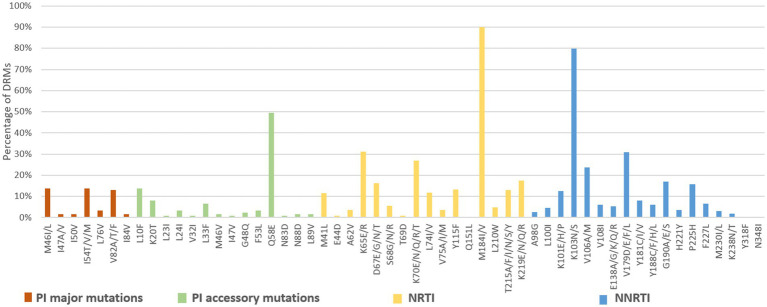
DRMs to the main ART classes among HIV patients with treatment failure in Liangshan Prefecture, China. PI, Protease inhibitor; NRTI, Nucleoside Reverse Transcriptase Inhibitor; NNRTI, Non-Nucleoside Reverse Transcriptase Inhibitor.

**Figure 7:**
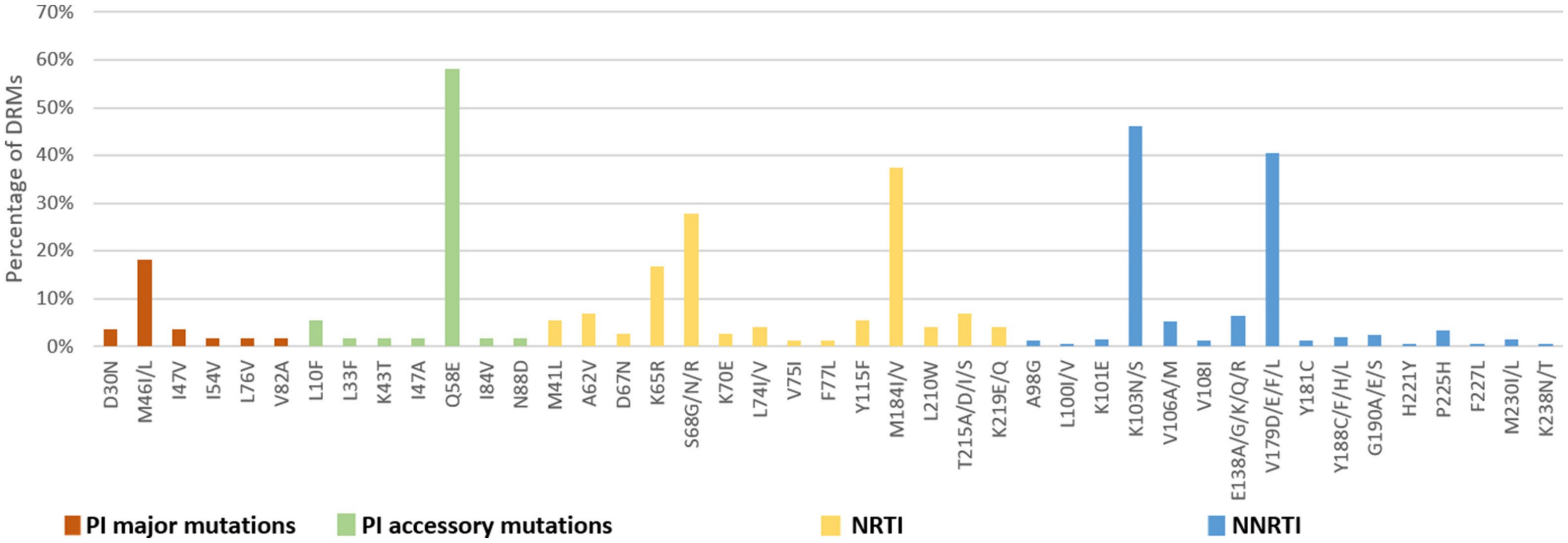
DRMs to the main ART classes among people newly diagnosed with HIV in Liangshan Prefecture, China. PI, Protease inhibitor; NRTI, Nucleoside Reverse Transcriptase Inhibitor; NNRTI, Non-Nucleoside Reverse Transcriptase Inhibitor.

Among the 3,924 successfully amplified samples from individuals with treatment failure, 80 NRTI-associated, 78 NNRTI-associated, and 43 PI-associated drug resistance mutations were identified (Figures 6, 7). The most prominent NRTI resistance mutations included M184I/V (89.9%), followed by K65E/R (31.2%), K70E/N/Q/R/T (26.9%), and K219E/N/Q/R (17.6%). The most common NNRTI-associated drug resistance mutations were K103N/S, accounting for 79.9%, followed by the V179D/E/F/L and V106A/M mutations, accounting for 30.9 and 23.7%, respectively. The most common PI-associated drug resistance mutations were Q58E (49.6%), followed by M46V (13.8%), I54T/V/M (13.8%) and L10F (13.8%; Figure 6).

The number of drug-resistance-associated mutations among newly diagnosed individuals was fewer than treatment failure individuals. 34 NRTI-associated, 56 NNRTI-associated, and 20 PI-associated drug resistance mutations were identified. The most common NRTIs mutations were M184I/V (37.5%) and S68G/N/R (27.8%). The most prominent NNRTIs resistance mutations included K103N/S (46.2%) and V179D/E/F/L (40.6%). The most common PI mutations were Q58E (58.2%), followed by M46I/L (18.2%; Figure 7).

Analysis of NRTI mutations showed that S68-associated mutations were detected in only 5.7% of treatment failure individuals but in 27.8% of newly diagnosed individuals. M184-associated mutations were present in 89.9% of treatment failure participants, but in 37.5% of newly diagnosed individuals. For NNRTI mutations, the proportions of G190-associated and V106-associated mutations in newly diagnosed individuals were smaller than treatment failure participants, which only occurred at frequencies of <6%. For PI variants, I54-associated and V82-associated mutations were observed in both treatment failure and newly diagnosed participants, though the proportion of newly diagnosed individuals with these mutations was significantly smaller (1.8% vs. 13.8 and 1.8% vs. 13.0%) than in treatment failure participants (Figures 6, 7).

### 3.5 Risk factors for drug resistance mutations

Univariable and multivariable logistic regression analyses were performed to identify factors associated with DRM. The results showed that in our population, gender was not a predictor of ADR [OR (95% CI): 0.93 (0.81–1.06)], whereas there was a negative association between the presence of ADR and individuals treated with NRTI+PI containing ART regimens [AOR (95% CI): 0.64 (0.45–0.89)], compared with NRTI+NNRTI based ART regimen (Table 3). Compared with heterosexual transmission, injecting drug use was a risk factor for ADR [AOR (95% CI): 1.29 (1.11–1.49); Table 3]. In multivariable logistic regression, all of the variables were not significantly associated with PI ADR (Table 4). In the multivariable logistic regression model of NRTI ADR, age, transmission route, marital status, and ART regimen were significantly associated with DRM (Table 5). In the multivariate model, the transmission route and ART regimen were independently associated with NNRTI ADR (Table 6). Compared with heterosexual transmission, injecting drug use had a higher prevalence of NNRTI ADR [AOR (95% CI): 1.29 (1.12–1.50)]. NNRTI ADR was found less frequently in patients receiving ART regimens with NRTI+PI [AOR (95% CI): 0.51 (0.36–0.72)] than in those with NRTI+NNRTI (Table 6).

**Table 3:** Risk factors for ADR among HIV patients with treatment failure in Liangshan Prefecture, China.

Variable	Total (*n* = 3,924)	ADR (*n* = 2,254)	Unadjusted OR	*p*-value	Adjusted OR	*p*-value
Time since diagnosis (years)						
1–5	1865	1,095 (58.7)	1			
6–10	1,136	652 (57.4)	0.95 (0.82–1.10)	0.48		
>10	923	507 (54.9)	0.86 (0.73–1.01)	0.06		
Gender						
Male	2,806	1,627 (57.9)	1			
Female	1,118	627 (56.1)	0.93 (0.81–1.06)	0.28		
Age (years)						
25–44	2,505	1,386 (55.3)	1		1	
<18	435	340 (75.1)	2.43 (1.94–3.05)	**<0.001**	1.15 (0.50–2.61)	0.74
18–24	152	77 (50.7)	0.83 (0.59–1.15)	0.26	0.79 (0.54–1.15)	0.22
45–64	709	405 (57.1)	1.08 (0.91–1.27)	0.40	1.08 (0.91–1.28)	0.38
≥ 65	105	46 (43.8)	0.63 (0.43–0.93)	**0.02**	0.68 (0.45–1.03)	0.07
Ethnicity						
Yi	3,621	2092 (57.8)	1			
Han	288	153 (53.1)	0.83 (0.65–1.05)	0.13		
Others	15	9 (60.0)	1.09 (0.39–3.09)	0.86		
Transmission route						
Heterosexual	1,688	871 (51.6)	1		1	
Injecting drug use	1,518	892 (58.8)	1.34 (1.16–1.54)	**<0.001**	1.29 (1.11–1.49)	**<0.001**
Mother to child transmission	352	264 (75.0)	2.81 (2.17–3.65)	**<0.001**	1.47 (0.73–2.94)	0.28
Men who have sex with men	17	11 (64.7)	1.72 (0.63–4.67)	0.29	1.65 (0.59–4.54)	0.34
Heterosexual +Injecting drug use	190	104 (54.7)	1.13 (0.84–1.53)	0.41	1.06 (0.79–1.44)	0.69
Uncertain	159	112 (70.4)	2.24 (1.57–3.18)	**<0.001**	1.33 (0.71–2.50)	0.38
Education						
Illiterate	2,246	1,300 (57.9)	1			
Primary school	1,320	752 (56.9)	0.96 (0.84–1.11)	0.59		
Junior high school	278	162 (58.3)	1.02 (0.79–1.31)	0.90		
High school or technical secondary school	65	33 (50.8)	0.75 (0.46–1.23)	0.25		
College or above	15	7 (46.7)	0.64 (0.23–1.76)	0.39		
Marital status						
Married or cohabiting	2060	1,127 (54.7)	1		1	
Unmarried	1,347	834 (61.9)	1.35 (1.17–1.55)	**<0.001**	1.01 (0.85–1.19)	0.92
Divorced or widowed	461	256 (55.5)	1.03 (0.84–1.27)	0.75	1.09 (0.89–1.35)	0.38
Unknown	56	37 (66.1)	1.61 (0.92–2.82)	0.09	1.55 (0.88–2.74)	0.13
Occupation						
Farmer	2,947	1,631 (55.3)	1		1	
Laborer	69	36 (52.2)	0.88 (0.55–1.42)	0.60	0.89 (0.55–1.44)	0.63
Unemployed	197	111 (56.3)	1.04 (0.78–1.39)	0.78	0.97 (0.72–1.31)	0.84
Government and public institutions	7	4 (57.1)	1.08 (0.24–4.82)	0.92	1.24 (0.27–5.76)	0.78
Business services	11	6 (54.5)	0.97 (0.29–3.18)	0.96	1.14 (0.34–3.83)	0.83
Children and students	481	359 (74.6)	2.37 (1.91–2.95)	**<0.001**	1.69 (0.71–3.99)	0.24
Others	212	107 (50.5)	0.82 (0.62–1.09)	0.17	0.76 (0.57–1.01)	0.06
HIV-1 variants						
CRF07_BC	3,752	2,156 (57.5)	1			
CRF01_AE	33	19 (57.6)	1.01 (0.50–2.01)	0.99		
CRF08_BC	104	58 (55.8)	0.93 (0.63–1.38)	0.73		
URFs	25	13 (52.0)	0.80 (0.37–1.76)	0.58		
Minor	10	8 (80.0)	2.96 (0.63–13.96)	0.17		
Recent CD4+ T lymphocyte count (pcs/μL)						
>350	2065	1,169 (56.6)	1			
200–350	1,134	659 (58.1)	1.09 (0.92–1.30)	0.29		
<200	717	422 (58.8)	1.06 (0.92–1.23)	0.41		
ART regimen at treatment failure						
NRTI+NNRTI	3,767	2,183 (58.0)	1		1	
NRTI+PI	152	68 (44.7)	0.59 (0.42–0.81)	**<0.001**	0.64 (0.45–0.89)	**0.01**
Triple NRTI	4	3 (75.0)	2.18 (0.23–20.95)	0.50	2.26 (0.22–22.95)	0.49
PI monotherapy	1	0	0	1	0	1

*Adjusted for: age, transmission route, marital status, occupation, ART regimen. ADR, Acquired drug resistance; NRTI, Nucleoside Reverse Transcriptase Inhibitor; NNRTI, Non-Nucleoside Reverse Transcriptase Inhibitor; PI, Protease inhibitor; URFs, Unique Recombinant Forms. *p*-values < 0.05 are displayed in bold.

**Table 4:** Risk factors for PI ADR among HIV patients with treatment failure in Liangshan Prefecture, China.

Variable	Total (*n* = 3,924)	PI ADR (*n* = 123)	Unadjusted OR	*p*-value	Adjusted OR	*p*-value
Time since diagnosis (years)						
1–5	1865	51 (2.7)	1			
6–10	1,136	35 (3.1)	1.13 (0.73–1.75)	0.58		
>10	923	37 (4.0)	1.48 (0.97–2.29)	0.07		
Gender						
Male	2,806	97 (3.5)	1			
Female	1,118	26 (2.3)	0.67 (0.43–1.03)	0.07		
Age (years)						
25–44	2,505	72 (2.9)	1		1	
<18	435	24 (5.5)	1.89 (1.18–3.03)	**0.01**	0.47 (0.07–3.04)	0.43
18–24	152	5 (3.3)	1.15 (0.46–2.89)	0.77	0.79 (0.23–2.66)	0.69
45–64	709	21 (3.0)	1.03 (0.63–1.69)	0.90	1.04 (0.64–1.71)	0.87
≥ 65	105	1 (1.0)	0.33 (0.05–2.36)	0.27	0.37 (0.05–2.69)	0.32
Ethnicity						
Yi	3,621	115 (3.2)	1			
Han	288	8 (2.8)	0.87 (0.42–1.80)	0.71		
Others	15	0	0			
Transmission route						
Heterosexual	1,688	42 (2.5)	1		1	
Injecting drug use	1,518	46 (3.0)	1.23 (0.80–1.87)	0.35	1.18 (0.76–1.82)	0.46
Mother to child transmission	352	22 (6.3)	2.61 (1.54–4.44)	**<0.001**	3.95 (0.84–18.59)	0.08
Men who have sex with men	17	0	0		0	
Heterosexual +Injecting drug use	190	7 (3.7)	1.49 (0.66–3.39)	0.33	1.42 (0.62–3.21)	0.41
Uncertain	159	6 (3.8)	1.54 (0.64–3.67)	0.33	2.08 (0.49–8.77)	0.32
Education						
Illiterate	2,246	77 (3.4)	1			
Primary school	1,320	37 (2.8)	0.81 (0.55–1.21)	0.31		
Junior high school	278	7 (2.5)	0.73 (0.33–1.59)	0.43		
High school or technical secondary school	65	1 (1.5)	0.44 (0.06–3.21)	0.42		
College or above	15	1 (6.7)	2.01 (0.26–15.49)	0.50		
Marital status						
Married or cohabiting	2060	59 (2.9)	1			
Unmarried	1,347	47 (3.5)	1.23 (0.83–1.81)	0.31		
Divorced or widowed	461	13 (2.8)	0.98 (0.54–1.81)	0.96		
Unknown	56	4 (7.1)	2.61 (0.91–7.45)	0.07		
Occupation						
Farmer	2,947	83 (2.8)	1		1	
Laborer	69	3 (4.3)	1.57 (0.48–5.09)	0.45	1.57 (0.48–5.13)	0.45
Unemployed	197	5 (2.5)	0.89 (0.60–2.24)	0.82	0.89 (0.36–2.25)	0.82
Government and public institutions	7	0	0		0	
Business services	11	0	0		0	
Children and students	481	26 (5.4)	1.97 (1.26–3.09)	**0.01**	1.29 (0.18–9.17)	0.79
Others	212	6 (2.8)	1.01 (0.43–2.33)	0.99	0.99 (0.43–2.32)	0.99
HIV-1 variants						
CRF07_BC	3,752	119 (3.2)	1			
CRF01_AE	33	0	0			
CRF08_BC	104	4 (3.8)	1.22 (0.44–3.37)	0.70		
URFs	25	0	0			
Minor	10	0	0			
Recent CD4+ T lymphocyte count (pcs/μL)						
>350	2065	61 (2.9)	1			
200–350	1,134	37 (3.3)	1.11 (0.73–1.68)	0.63		
<200	717	25 (3.5)	1.19 (0.74–1.91)	0.49		
ART regimen at treatment failure						
NRTI+NNRTI	3,767	119 (3.2)	1			
NRTI+PI	152	4 (2.6)	0.83 (0.30–2.27)	0.72		
Triple NRTI	4	0	0			
PI monotherapy	1	0	0			

*Adjusted for: age, transmission route, occupation. ADR, Acquired drug resistance; NRTI, Nucleoside Reverse Transcriptase Inhibitor; NNRTI, Non-Nucleoside Reverse Transcriptase Inhibitor; PI, Protease inhibitor; URFs, Unique Recombinant Forms. *p*-values < 0.05 are displayed in bold.

**Table 5:** Risk factors for NRTI ADR among HIV patients with treatment failure in Liangshan Prefecture, China.

Variable	Total (*n* = 3,924)	NRTI ADR (*n* = 934)	Unadjusted OR	*p*-value	Adjusted OR	*p*-value
Time since diagnosis (years)						
1–5	1865	458 (24.6)	1			
6–10	1,136	261 (22.9)	0.92 (0.77–1.09)	0.33		
>10	923	215 (23.3)	0.93 (0.78–1.12)	0.46		
Gender						
Male	2,806	619 (22.1)	1		1	
Female	1,118	315 (28.2)	1.39 (1.18–1.62)	**<0.001**	1.16 (0.96–1.39)	0.12
Age (years)						
25–44	2,505	481 (19.2)	1		1	
<18	435	237 (54.5)	4.62 (3.74–5.69)	**<0.001**	0.87 (0.36–2.09)	0.75
18–24	152	28 (18.4)	0.95 (0.62–1.45)	0.81	0.53 (0.31–0.92)	**0.02**
45–64	709	173 (24.4)	1.36 (1.11–1.66)	**0.01**	1.33 (1.08–1.63)	**0.01**
≥ 65	105	15 (14.3)	0.70 (0.40–1.22)	0.21	0.66 (0.37–1.17)	0.16
Ethnicity						
Yi	3,621	869 (23.9)	1			
Han	288	61 (21.2)	0.85 (0.64–1.14)	0.28		
Others	15	4 (26.7)	1.15 (0.37–3.63)	0.81		
Transmission route						
Heterosexual	1,688	375 (22.2)	1		1	
Injecting drug use	1,518	261 (17.2)	0.73 (0.61–0.87)	**<0.001**	0.73 (0.59–0.88)	**<0.001**
Mother to child transmission	352	190 (53.9)	4.11 (3.23–5.22)	**<0.001**	2.12 (1.06–4.54)	**0.04**
Men who have sex with men	17	4 (23.5)	1.08 (0.35–3.32)	0.89	0.93 (0.28–3.05)	0.90
Heterosexual +Injecting drug use	190	37 (19.5)	0.85 (0.58–1.23)	0.39	0.81 (0.55–1.20)	029
Uncertain	159	67 (42.1)	2.55 (1.82–3.57)	**<0.001**	1.53 (0.78–3.02)	0.22
Education						
Illiterate	2,246	568 (25.3)	1		1	
Primary school	1,320	286 (21.7)	0.82 (0.69–0.96)	**0.01**	0.98 (0.83–1.17)	0.82
Junior high school	278	60 (21.6)	0.81 (0.60–1.09)	0.19	1.13 (0.83–1.56)	0.44
High school or technical secondary school	65	16 (24.6)	0.97 (0.54–1.71)	0.90	1.47 (0.79–2.71)	0.22
College or above	15	4 (26.7)	1.07 (0.34–3.39)	0.90	1.54 (0.42–5.66)	0.51
Marital status						
Married or cohabiting	2060	434 (21.1)	1		1	
Unmarried	1,347	413 (30.6)	1.66 (1.42–1.94)	**<0.001**	1.03 (0.83–1.28)	0.77
Divorced or widowed	461	73 (15.8)	0.71 (0.54–0.93)	**0.01**	0.69 (0.52–0.90)	**0.01**
Unknown	56	14 (25)	1.25 (0.68–2.31)	0.48	1.55 (0.83–2.91)	0.17
Occupation						
Farmer	2,947	605 (20.5)	1		1	
Laborer	69	16 (23.2)	1.17 (0.66–2.06)	0.59	1.06 (0.59–1.88)	0.86
Unemployed	197	29 (14.7)	0.67 (0.45–1.00)	0.05	0.66 (0.43–1.01)	0.05
Government and public institutions	7	2 (28.6)	1.55 (0.30–8.00)	0.60	1.25 (0.19–7.85)	0.82
Business services	11	1 (9.1)	0.39 (0.05–3.03)	0.37	0.36 (0.05–2.89)	0.34
Children and students	481	248 (51.6)	4.12 (3.37–5.03)	**<0.001**	2.09 (0.84–5.19)	0.11
Others	212	33 (15.6)	0.71 (0.49–1.05)	0.08	0.73 (0.49–1.07)	0.11
HIV-1 variants						
CRF07_BC	3,752	897 (23.9)	1			
CRF01_AE	33	9 (27.3)	1.19 (0.55–2.58)	0.65		
CRF08_BC	104	21 (20.2)	0.81 (0.49–1.31)	0.38		
URFs	25	4 (16.0)	0.61 (0.21–1.77)	0.36		
Minor	10	3 (30.0)	1.36 (0.35–5.29)	0.65		
Recent CD4+ T lymphocyte count (pcs/μL)						
>350	2065	485 (23.5)	1			
200–350	1,134	270 (23.8)	1.02 (0.86–1.23)	0.84		
<200	717	178 (24.8)	1.08 (0.88–1.31)	0.47		
ART regimen						
NRTI+NNRTI	3,767	909 (24.1)	1		1	
NRTI+PI	152	23 (15.1)	0.56 (0.36–0.88)	**0.01**	0.54 (0.34–0.87)	**0.01**
Triple NRTI	4	2 (50.0)	3.14 (0.44–22.35)	0.25	3.12 (0.38–25.37)	0.29
PI monotherapy	1	0	0	1	0	1

*Adjusted for: gender, age, transmission route, marital status, occupation, ART regimen. ADR, Acquired drug resistance; NRTI, Nucleoside Reverse Transcriptase Inhibitor; NNRTI, Non-Nucleoside Reverse Transcriptase Inhibitor; PI, Protease inhibitor; URFs, Unique Recombinant Forms. *p*-values < 0.05 are displayed in bold.

**Table 6:** Risk factors for NNRTI ADR among HIV patients with treatment failure in Liangshan Prefecture, China.

Variable	Total (*n* = 3,924)	NNRTI ADR (*n* = 2,156)	Unadjusted OR	*p*-value	Adjusted OR	*p*-value
Time since diagnosis (years)						
1–5	1865	1,049 (56.2)	1			
6–10	1,136	622 (54.8)	0.94 (0.81–1.09)	0.43		
>10	923	485 (52.5)	0.86 (0.74–1.01)	0.07		
Gender						
Male	2,806	1,565 (55.8)	1			
Female	1,118	591 (52.9)	0.89 (0.77–1.02)	0.09		
Age (years)						
25–44	2,505	1,325 (52.9)	1		1	
<18	435	327 (75.2)	2.31 (1.86–2.88)	**<0.001**	1.12 (0.49–2.54)	0.78
18–24	152	76 (50.0)	0.89 (0.64–1.24)	0.49	0.87 (0.59–1.27)	0.48
45–64	709	384 (54.2)	1.05 (0.89–1.24)	0.55	1.06 (0.89–1.26)	0.53
≥ 65	105	44 (41.9)	0.64 (0.43–0.95)	**0.03**	0.72 (0.46–1.08)	0.11
Ethnicity						
Yi	3,621	2004 (55.3)	1			
Han	288	143 (49.7)	0.79 (0.63–1.01)	0.06		
Others	15	9 (60.0)	1.21 (0.43–3.41)	0.72		
Transmission route						
Heterosexual	1,688	825 (48.9)	1		1	
Injecting drug use	1,518	857 (56.5)	1.36 (1.18–1.56)	**<0.001**	1.29 (1.12–1.50)	**<0.001**
Mother to child transmission	352	255 (72.4)	2.75 (2.14–3.54)	**<0.001**	1.45 (0.73–2.89)	0.29
Men who have sex with men	17	11 (64.7)	1.92 (0.71–5.21)	0.20	1.79 (0.65–4.94)	0.26
Heterosexual +Injecting drug use	190	101 (53.2)	1.19 (0.88–1.60)	0.26	1.12 (0.82–1.51)	0.48
Uncertain	159	107 (67.3)	2.15 (1.53–3.04)	**<0.001**	1.29 (0.69–2.41)	0.43
Education						
Illiterate	2,246	1,239 (55.2)	1			
Primary school	1,320	721 (54.6)	0.98 (0.85–1.12)	0.75		
Junior high school	278	157 (56.5)	1.06 (0.82–1.36)	0.68		
High school or technical secondary school	65	33 (50.8)	0.84 (0.51–1.37)	0.48		
College or above	15	6 (40.0)	0.54 (0.19–1.53)	0.25		
Marital status						
Married or cohabiting	2060	1,077 (52.3)	1		1	
Unmarried	1,347	802 (59.5)	1.34 (1.17–1.54)	**<0.001**	0.99 (0.84–1.18)	0.95
Divorced or widowed	461	243 (52.7)	1.02 (0.83–1.25)	0.87	1.08 (0.88–1.32)	0.49
Unknown	56	34 (60.7)	1.41 (0.82–2.43)	0.22	1.32 (0.76–2.29)	0.32
Occupation						
Farmer	2,947	1,553 (52.7)	1		1	
Laborer	69	34 (49.3)	0.87 (0.54–1.41)	0.57	0.89 (0.55–1.43)	0.62
Unemployed	197	110 (55.8)	1.14 (0.85–1.52)	0.39	1.07 (0.79–1.44)	0.66
Government and public institutions	7	4 (57.1)	1.19 (0.27–5.36)	0.81	1.41 (0.30–6.59)	0.66
Business services	11	5 (45.5)	0.75 (0.23–2.46)	0.63	0.86 (0.26–2.89)	0.81
Children and students	481	346 (71.9)	2.30 (1.86–2.84)	**<0.001**	1.71 (0.73–4.03)	0.22
Others	212	104 (49.1)	0.86 (0.65–1.14)	0.31	0.80 (0.60–1.07)	0.13
HIV-1 variants						
CRF07_BC	3,752	2061 (54.9)	1			
CRF01_AE	33	19 (57.6)	1.11 (0.56–2.23)	0.76		
CRF08_BC	104	55 (52.9)	0.92 (0.62–1.36)	0.68		
URFs	25	13 (52.0)	0.89 (0.41–1.95)	0.77		
Minor	10	8 (80.0)	3.28 (0.69–15.48)	0.13		
Recent CD4+ T lymphocyte count (pcs/μL)						
>350	2065	1,123 (54.4)	1			
200–350	1,134	626 (55.2)	1.03 (0.89–1.19)	0.66		
<200	717	403 (56.2)	1.08 (0.91–1.28)	0.39		
ART regimen						
NRTI+NNRTI	3,767	2096 (55.6)	1		1	
NRTI+PI	152	57 (37.5)	0.48 (0.34–0.67)	**<0.001**	0.51 (0.36–0.72)	**<0.001**
Triple NRTI	4	3 (75.0)	2.39 (025–23.01)	0.45	2.44 (0.24–24.59)	0.45
PI monotherapy	1	0	0	1	0	1

*Adjusted for: age, transmission route, marital status, occupation, ART regimen. ADR, Acquired drug resistance; NRTI, Nucleoside Reverse Transcriptase Inhibitor; NNRTI, Non-Nucleoside Reverse Transcriptase Inhibitor; PI, Protease inhibitor; URFs, Unique Recombinant Forms. *p*-values < 0.05 are displayed in bold.

In multivariate analysis of PDR (Table 7), factors that were significantly associated with PDR were age ≥ 65 years [AOR (95% CI): 0.55 (0.31–0.97)] compared to age 25–44 years, HIV-1 minor variants [AOR (95% CI): 4.35 (1.78–10.64)] compared CRF07_BC, recent CD4+ T lymphocyte count 200–350 pcs/μl [AOR (95% CI): 1.80 (1.31–2.49)] and <200 pcs/μl [AOR (95% CI): 1.34 (1.05–1.71)], compared with >350 pcs/μl. Gender, ethnicity, transmission route, education, marital status, occupation and HIV-1 variants were not significantly associated with PDR. Multivariate logistic regression analysis revealed that transmission route and occupation were the most significant factors associated with PI PDR (Table 8). Mother to child transmission [AOR (95% CI): 0.06 (0.01–0.98)] was associated with PI PDR compared to heterosexual transmission, while unemployment [AOR (95% CI): 3.69 (1.06–12.81)] and children and students [AOR (95% CI): 7.79 (1.16–52.61)] were associated with a higher risk of PI PDR, compared to farmers. In the multivariate model, the following four factors were independently correlated with NRTI PDR (Table 9): other ethnicity [AOR (95% CI): 3.92 (1.11–13.91)] had higher risk than Yi ethnicity, the rate of NRTI PDR among high school or technical secondary school [AOR (95% CI): 3.55 (1.34–9.43)] was higher than illiterate people, unmarried [AOR (95% CI): 1.95 (1.10–3.46)] had higher risk than married or cohabiting people, and HIV-1 variant CRF01_AE [AOR (95% CI): 3.99 (1.65–9.67)] was associated with a higher risk than CRF07_BC. Some of the factors that were independently associated with an increased risk of NNRTI PDR (Table 10), included education, HIV-1 variants and recent CD4+ T lymphocyte count. Primary school education [AOR (95% CI): 0.76 (0.59–0.98)] was associated with a lower risk than illiterate people, minor HIV-1 variants [AOR (95% CI): 5.13 (2.11–12.48)] with higher risk than CRF07_BC, and recent CD4+ T lymphocyte count<200 pcs/μl [AOR (95% CI): 1.53 (1.09–2.15)] with higher risk than>350 pcs/μl of NNRTI PDR.

**Table 7:** Risk factors to PDR among people newly diagnosed with HIV in Liangshan Prefecture, China.

Variable	Total (*n* = 1999)	PDR (*n* = 478)	Unadjusted OR	*p*-value	Adjusted OR	*p*-value
Gender						
Male	1,070	247 (23.1)	1			
Female	929	231 (24.9)	1.10 (0.89–1.36)	0.35		
Age (years)						
25–44	1,011	248 (24.5)	1		1	
<18	207	65 (31.4)	1.41 (1.02–1.95)	**0.04**	1.68 (0.70–4.01)	0.24
18–24	178	39 (21.9)	0.86 (0.59–1.27)	0.45	0.84 (0.56–1.27)	0.41
45–64	501	107 (21.4)	0.84 (0.65–1.08)	0.17	0.81 (0.62–1.06)	0.13
≥ 65	102	19 (18.6)	0.70 (0.42–1.18)	0.19	0.55 (0.31–0.97)	**0.04**
Ethnicity						
Yi	1705	411 (24.1)	1			
Han	271	60 (22.1)	0.89 (0.66–1.22)	0.48		
Others	23	7 (30.4)	1.38 (0.56–3.37)	0.48		
Transmission route						
Heterosexual	1734	402 (23.2)	1			
Injecting drug use	37	12 (32.4)	1.59 (0.79–3.19)	0.19		
Mother to child transmission	83	25 (30.1)	1.43 (0.88–2.31)	0.15		
Men who have sex with men	13	4 (30.8)	1.47 (0.45–4.81)	0.52		
Heterosexual +Injecting drug use	12	1 (8.3)	0.30 (0.04–2.34)	0.25		
Uncertain	120	34 (28.3)	1.31 (0.87–1.98)	0.19		
Education						
Illiterate	917	230 (25.1)	1			
Primary school	776	173 (22.3)	0.86 (0.68–1.07)	0.18		
Junior high school	204	51 (25.0)	0.99 (0.70–1.41)	0.98		
High school or technical secondary school	52	14 (26.9)	1.10 (0.59–2.07)	0.77		
College or above	50	10 (20.0)	0.75 (0.37–1.52)	0.42		
Marital status						
Married or cohabiting	870	181 (20.8)	1		1	
Unmarried	568	157 (27.6)	1.45 (1.14–1.86)	**0.01**	1.28 (0.93–1.75)	0.13
Divorced or widowed	556	138 (24.8)	1.26 (0.98–1.62)	0.08	1.32 (1.02–1.735)	**0.04**
Unknown	5	2 (40.0)	2.54 (0.42–15.30)	0.31	1.79 (0.18–18.18)	0.62
Occupation						
Farmer	1,674	389 (23.2)	1		1	
Laborer	25	4 (16.0)	0.63 (0.22–1.84)	0.39	0.56 (0.19–1.65)	0.29
Unemployed	36	12 (33.3)	1.65 (0.82–3.33)	0.16	1.67 (0.79–3.50)	0.17
Government and public institutions	31	7 (22.6)	0.96 (0.41–2.25)	0.93	0.99 (0.39–2.48)	0.99
Business services	10	1 (10.0)	0.37 (0.05–2.91)	0.34	0.32 (0.04–2.52)	0.28
Children and students	189	58 (30.7)	1.46 (1.05–2.03)	**0.02**	0.69 (0.28–1.66)	0.40
Others	34	7 (20.6)	0.86 (0.37–1.98)	0.72	0.94 (0.39–2.23)	0.88
HIV-1 variants						
CRF07_BC	1817	425 (23.4)	1		1	
CRF01_AE	50	15 (30.0)	1.40 (0.76–2.59)	0.28	1.46 (0.76–2.82)	0.26
CRF08_BC	80	17 (21.2)	0.88 (0.51–1.53)	0.66	0.99 (0.57–1.75)	0.99
URFs	31	10 (32.3)	1.56 (0.73–3.34)	0.25	1.51 (0.68–3.31)	0.31
Minor	21	11 (52.4)	3.60 (1.52–8.54)	**<0.01**	4.35 (1.78–10.64)	**<0.001**
Recent CD4+ T lymphocyte count (pcs/μL)						
>350	1,162	250 (21.5)	1		1	
200–350	511	134 (26.2)	1.29 (1.02–1.65)	**0.04**	1.34 (1.05–1.71)	**0.02**
<200	218	70 (32.1)	1.73 (1.26–2.37)	**<0.001**	1.80 (1.31–2.49)	**<0.001**

*Adjusted for: age, marital status, occupation, HIV-1 variants, CD4+ T lymphocyte count. PDR, Pre-treatment drug resistance; URFs, Unique Recombinant Forms. *p*-values < 0.05 are displayed in bold.

**Table 8:** Risk factors for PI PDR among people newly diagnosed with HIV in Liangshan Prefecture, China.

Variable	Total (*n* = 1999)	PI PDR (*n* = 55)	Unadjusted OR	*p*-value	Adjusted OR	*p*-value
Gender						
Male	1,070	33 (60.0)	1			
Female	929	22 (40.0)	0.76 (0.44–1.32)	0.33		
Age (years)						
25–44	1,011	10 (18.2)	1			
<18	207	4 (7.3)	1.66 (0.79–3.45)	0.18		
18–24	178	30 (54.5)	0.75 (0.26–2.16)	0.59		
45–64	501	10 (18.2)	0.67 (0.32–1.37)	0.27		
≥ 65	102	1 (1.8)	0.32 (0.04–2.39)	0.27		
Ethnicity						
Yi	1705	45 (81.8)	1			
Han	271	10 (18.2)	1.41 (0.70–2.84)	0.33		
Others	23	0	0			
Transmission route						
Heterosexual	1734	44 (80.0)	1		1	
Injecting drug use	37	1 (1.8)	1.07 (0.14–7.96)	0.95	1.11 (0.15–8.36)	0.92
Mother to child transmission	83	1 (1.8)	0.47 (0.06–3.44)	0.46	0.06 (0.01–0.98)	**0.04**
Men who have sex with men	13	1 (1.8)	3.20 (0.41–25.16)	0.27	1.33 (0.14–12.83)	0.81
Heterosexual +Injecting drug use	12	0	0		0	
Uncertain	120	8 (14.5)	2.74 (1.26–5.97)	**0.01**	0.44 (0.06–3.17)	0.41
Education						
Illiterate	917	21 (38.2)	1			
Primary school	776	21 (38.2)	1.19 (0.64–2.19)	0.58		
Junior high school	204	8 (14.5)	1.74 (0.76–3.99)	0.19		
High school or technical secondary school	52	2 (3.6)	1.71 (0.39–7.48)	0.48		
College or above	50	3 (5.5)	2.72 (0.78–9.46)	0.12		
Marital status						
Married or cohabiting	870	21 (38.2)	1			
Unmarried	568	20 (36.4)	1.48 (0.79–2.75)	0.22		
Divorced or widowed	556	14 (25.5)	1.04 (0.53–2.07)	0.90		
Unknown	5	0	0			
Occupation						
Farmer	1,674	40 (72.7)	1		1	
Laborer	25	0	0		0	
Unemployed	36	3 (5.5)	3.71 (1.09–12.61)	**0.04**	3.69 (1.06–12.81)	**0.04**
Government and public institutions	31	0	0		0	
Business services	10	0	0		0	
Children and students	189	10 (18.2)	2.28 (1.12–4.64)	**0.02**	7.79 (1.16–52.61)	**0.04**
Others	34	2 (3.6)	2.55 (0.59–11.02)	0.21	2.64 (0.61–11.49)	0.19
HIV-1 variants						
CRF07_BC	1817	49 (89.1)	1			
CRF01_AE	50	3 (5.5)	2.30 (0.69–7.66)	0.17		
CRF08_BC	80	1 (1.8)	0.46 (0.06–3.35)	0.44		
URFs	31	2 (3.6)	2.49 (0.58–10.72)	0.22		
Minor	21	0	0			
Recent CD4+ T lymphocyte count (pcs/μL)						
>350	1,162	9 (16.4)	1			
200–350	511	15 (27.3)	1.27 (0.67–2.41)	0.46		
<200	218	27 (49.1)	1.81 (0.84–3.90)	0.13		

*Adjusted for: transmission route, occupation. PDR, Pre-treatment drug resistance; PI, Protease inhibitor; URFs, Unique Recombinant Forms. *p*-values < 0.05 are displayed in bold.

**Table 9:** Risk factors for NRTI PDR among people newly diagnosed with HIV in Liangshan Prefecture, China.

Variable	Total (*n* = 1999)	NRTI PDR (*n* = 72)	Unadjusted OR	*p*-value	Adjusted OR	*p*-value
Gender						
Male	1,070	39 (2.6)	1			
Female	929	33 (2.6)	0.97 (0.61–1.56)	0.91		
Age (years)						
25–44	1,011	30 (2.9)	1			
<18	207	10 (4.8)	1.66 (0.79–3.45)	0.18		
18–24	178	10 (5.6)	1.95 (0.93–4.06)	0.08		
45–64	501	21 (4.2)	1.43 (0.81–2.53)	0.22		
≥ 65	102	1 (1.0)	0.32 (0.04–2.39)	0.27		
Ethnicity						
Yi	1705	60 (3.5)	1		1	
Han	271	9 (3.3)	0.94 (0.46–1.92)	0.87	0.78 (0.36–1.67)	0.52
Others	23	3 (13.0)	4.11 (1.19–14.22)	**0.03**	3.92 (1.11–13.91)	**0.03**
Transmission route						
Heterosexual	1734	61 (3.5)	1			
Injecting drug use	37	1 (2.7)	0.76 (0.10–5.65)	0.79		
Mother to child transmission	83	5 (6.0)	1.76 (0.69–4.49)	0.24		
Men who have sex with men	13	0	0			
Heterosexual +Injecting drug use	12	0	0			
Uncertain	120	5 (4.2)	1.19 (0.47–3.03)	0.71		
Education						
Illiterate	917	29 (3.2)	1		1	
Primary school	776	26 (3.4)	1.06 (0.62–1.82)	0.83	1.05 (0.61–1.81)	0.87
Junior high school	204	8 (3.9)	1.25 (0.56–2.78)	0.58	1.12 (0.49–2.59)	0.79
High school or technical secondary school	52	6 (11.5)	3.99 (1.58–10.09)	**0.01**	3.55 (1.34–9.43)	**0.01**
College or above	50	3 (6.0)	1.96 (0.58–6.65)	0.28	1.51 (0.41–5.52)	0.54
Marital status						
Married or cohabiting	870	22 (2.5)	1		1	
Unmarried	568	30 (5.2)	2.15 (1.23–3.77)	**0.01**	1.95 (1.10–3.46)	**0.02**
Divorced or widowed	556	20 (3.6)	1.44 (0.78–2.66)	0.25	1.42 (0.76–2.63)	0.28
Unknown	5	0	0		0	
Occupation						
Farmer	1,674	57 (3.4)	1			
Laborer	25	0	0			
Unemployed	36	4 (11.1)	3.55 (1.21–10.36)	0.02		
Government and public institutions	31	2 (6.5)	1.96 (0.46–8.39)	0.37		
Business services	10	0	0			
Children and students	189	9 (4.8)	1.42 (0.69–2.91)	0.34		
Others	34	0	0			
HIV-1 variants						
CRF07_BC	1817	58 (3.2)	1		1	
CRF01_AE	50	7 (14.0)	4.94 (2.13–11.44)	**<0.001**	3.99 (1.65–9.67)	**<0.01**
CRF08_BC	80	4 (5.0)	1.59 (0.57–4.51)	0.38	1.56 (0.54–4.49)	0.41
URFs	31	3 (9.7)	3.25 (0.96–10.99)	0.06	3.38 (0.98–11.61)	0.05
Minor	21	0	0		0	
Recent CD4+ T lymphocyte count (pcs/μL)						
>350	1,162	34 (2.9)	1			
200–350	511	23 (4.5)	1.56 (0.91–2.68)	0.11		
<200	218	12 (5.5)	1.93 (0.98–3.79)	0.56		

*Adjusted for: ethnicity, education, marital status, HIV-1 variants. NRTI, Nucleoside Reverse Transcriptase Inhibitor; PDR, Pre-treatment drug resistance; URFs, Unique Recombinant Forms. *p*-values < 0.05 are displayed in bold.

**Table 10:** Risk factors for NNRTI PDR among people newly diagnosed with HIV in Liangshan Prefecture, China.

Variable	Total (*n* = 1999)	NNRTI PDR (*n* = 409)	Unadjusted OR	*p*-value	Adjusted OR	*p*-value
Gender						
Male	1,070	210 (19.6)	1			
Female	929	199 (21.4)	1.12 (0.89–1.39)	0.32		
Age (years)						
25–44	1,011	216 (21.4)	1			
<18	207	56 (27.1)	1.37 (0.97–1.92)	0.07		
18–24	178	30 (16.9)	0.75 (0.49–1.14)	0.17		
45–64	501	89 (17.8)	0.79 (0.60–1.05)	0.10		
≥ 65	102	18 (17.6)	0.79 (0.46–1.34)	0.38		
Ethnicity						
Yi	1705	354 (20.8)	1			
Han	271	49 (18.1)	0.84 (0.61–1.17)	0.31		
Others	23	6 (26.1)	1.35 (0.53–3.44)	0.53		
Transmission route						
Heterosexual	1734	343 (19.8)	1			
Injecting drug use	37	11 (29.7)	1.72 (0.84–3.51)	0.14		
Mother to child transmission	83	23 (27.7)	1.56 (0.95–2.55)	0.08		
Men who have sex with men	13	3 (23.1)	1.22 (0.33–4.45)	0.77		
Heterosexual +Injecting drug use	12	1 (8.3)	0.37 (0.05–2.87)	0.34		
Uncertain	120	28 (23.3)	1.23 (0.79–1.92)	0.35		
Education						
Illiterate	917	207 (22.6)	1		1	
Primary school	776	144 (18.6)	0.78 (0.62–0.99)	**0.04**	0.76 (0.59–0.98)	**0.03**
Junior high school	204	43 (21.1)	0.92 (0.63–1.33)	0.64	0.82 (0.55–1.21)	0.31
High school or technical secondary school	52	9 (17.3)	0.72 (0.34–1.49)	0.38	0.72 (0.34–1.52)	0.39
College or above	50	6 (12)	0.47 (0.19–1.11)	0.09	0.42 (0.17–1.04)	0.06
Marital status						
Married or cohabiting	870	159 (18.3)	1		1	
Unmarried	568	129 (22.7)	1.31 (1.01–1.71)	**0.04**	1.29 (0.99–1.71)	0.06
Divorced or widowed	556	119 (21.4)	1.22 (0.93–1.59)	0.15	1.19 (0.90–1.57)	0.21
Unknown	5	2 (40.0)	2.98 (0.49–17.99)	0.23	1.76 (0.18–17.09)	0.63
Occupation						
Farmer	1,674	338 (20.2)	1			
Laborer	25	4 (16.0)	0.75 (0.26–2.21)	0.61		
Unemployed	36	6 (16.7)	0.79 (0.33–1.92)	0.60		
Government and public institutions	31	6 (19.4)	0.95 (0.39–2.33)	0.91		
Business services	10	1 (10.0)	0.44 (0.06–3.48)	0.44		
Children and students	189	49 (25.9)	1.38 (0.98–1.96)	0.07		
Others	34	5 (14.7)	0.68 (0.26–1.77)	0.43		
HIV-1 variants						
CRF07_BC	1817	366 (89.5)	1		1	
CRF01_AE	50	9 (2.2)	0.87 (0.42–1.81)	0.71	0.89 (0.41–1.96)	0.78
CRF08_BC	80	13 (3.2)	0.77 (0.42–1.41)	0.39	0.88 (0.47–1.61)	0.69
URFs	31	10 (2.4)	1.89 (0.88–4.04)	0.10	1.94 (0.88–4.24)	0.09
Minor	21	11 (2.7)	1.36 (1.84–10.35)	**<0.001**	5.13 (2.11–12.48)	**<0.001**
Recent CD4+ T lymphocyte count (pcs/μL)						
>350	1,162	217 (18.7)	1		1	
200–350	511	113 (22.1)	1.24 (0.96–1.59)	0.10	1.25 (0.96–1.62)	0.09
<200	218	58 (26.6)	1.58 (1.13–2.21)	**0.01**	1.53 (1.09–2.15)	**0.01**

*Adjusted for: education, marital status, HIV-1 variants, CD4+ T lymphocyte count. NNRTI, Non-Nucleoside Reverse Transcriptase Inhibitor; PDR, Pre-treatment drug resistance; URFs, Unique Recombinant Forms. *p*-values < 0.05 are displayed in bold.

### 3.6 Trends of drug resistance mutations

**Figure 8:**
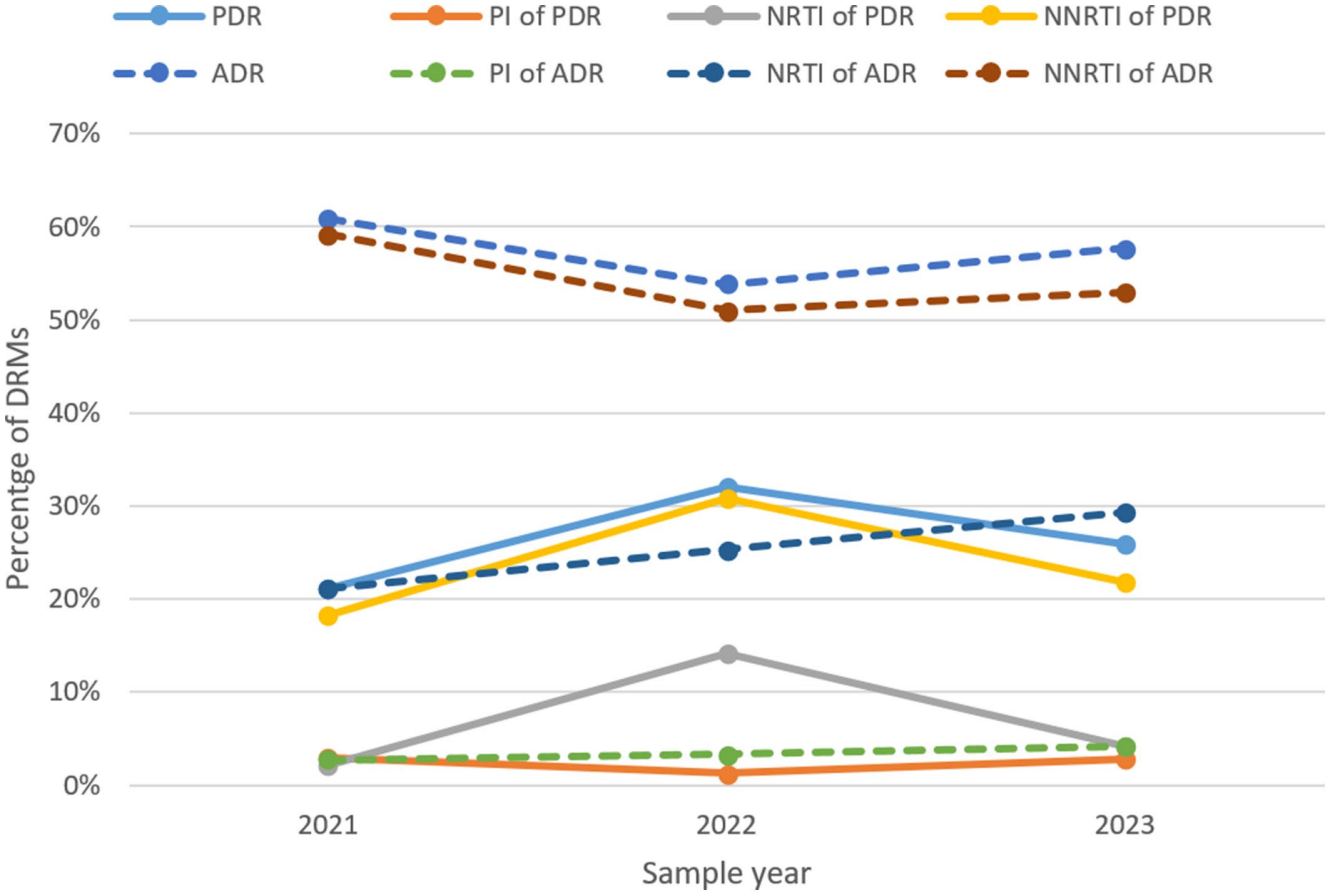
The trend of prevalence of DRMs in Liangshan Prefecture, China. ADR, acquired drug resistance; PDR, pre-treatment drug resistance; PI, Protease inhibitor; NRTI, Nucleoside Reverse Transcriptase Inhibitor; NNRTI, Non-Nucleoside Reverse Transcriptase Inhibitor; DRM, Drug Resistance Mutation.

The prevalence of ADR initially decreased from 60.9% in 2021 to 53.8% in 2022, but then increased to 57.7% in 2023, whereas PDR rose sharply from 21.1 to 32.1%, before declining to 25.9% (Figure 8; Table 11). Trends for NNRTI ADR and NNRTI PDR closely matched the overall trends of ADR and PDR. The prevalence of ADR and PDR associated with PI remained at low levels throughout the study period. PDR linked to NRTI increased from 2.1 to 14.1%, but then declined back to 4.1%. In contrast, ADR associated with NRTI displayed a continuous upward trend from 21.1% in 2021 to 29.3% in 2023.

**Table 11:** The occurrence of drug resistance to ART classes over the years in Liangshan Prefecture, China.

Year	PDR (%)	PI of PDR (%)	NRTI of PDR (%)	NNRTI of PDR (%)	ADR (%)	PI of ADR (%)	NRTI of ADR (%)	NNRTI of ADR (%)
2021	21.1	2.9	2.1	18.2	60.9	2.7	21.1	59.2
2022	32.1	1.3	14.1	30.8	53.8	3.3	25.3	51.0
2023	25.9	2.8	4.1	21.8	57.7	4.2	29.3	53.0

ADR, Acquired drug resistance; PDR, Pre-treatment drug resistance; PI, Protease inhibitor; NRTI, Nucleoside Reverse Transcriptase Inhibitor; NNRTI, Non-Nucleoside Reverse Transcriptase Inhibitor.

## 4 Discussion

In this study, we used a large sample size and the latest data to investigate HIV-1 drug resistance in Liangshan Prefecture for the first time. The proportion of males in newly diagnosed individuals was lower than other newly diagnosed groups in China (36–38), which showed an increasing trend of HIV infection in females in Liangshan. For transmission routes, the proportion of sexual transmission in the newly diagnosed individuals is significantly higher than in the treatment failure patients. Compared to treatment failure patients, the proportion of CRF07_BC in newly diagnosed individuals was lower, while other HIV-1 variants were more common. Our study showed a more widespread distribution of HIV-1 genotypes and that the heterosexual route comprised the predominant transmission route in Liangshan Prefecture. The overall prevalence of ADR and PDR was significantly higher in Liangshan Prefecture, compared to the overall situation in China (34, 39). These findings provide important guidance for the adjustment of future HIV treatment regimens and contribute to the body of literature on drug resistance.

ADR prevalence (57.4%) was lower than other treatment failure cohorts in China and other countries (40–42). This may be because of the government’s increased focus on ART treatment adherence in Liangshan Prefecture in recent years, accompanied by free treatment provision strategies. The overall prevalence of PDR was higher than in other studies in China and other countries (20, 38, 43–45) and also higher than in a previous study in Liangshan (37). According to the WHO criteria, PDR prevalence of below 5%, 5–15%, and above 15% are considered low, moderate, and high levels of HIV-1 drug resistance (46). The PDR prevalence in Liangshan Prefecture was relatively high (23.9%), highlighting the need to pay more attention to treating newly diagnosed individuals. The proportion of NNRTI PDR, NRTI PDR, and PI PDR is higher than in other studies of newly diagnosed individuals in China (36, 38, 44). The results showed that DRMs against NNRTIs and NRTIs were the main DRMs with high-level drug resistance in both treatment failure patients and newly diagnosed individuals, signifying a substantial concern for dual-class resistance.

Consistent with studies conducted in other areas of China (40), the prevalence of ADR to NNRTIs was substantially higher than that of NRTIs and PIs among patients with ART failure in Liangshan Prefecture. In this study, the main regimens were TDF + 3TC + NVP/EFV or AZT + 3TC + NVP/EFV, which account for more than 95.0%. Under the pressure of drug selection, the prevalence of ADR and PDR in Liangshan Prefecture was mainly driven by NNRTI-associated DRMs. The top NNRTI-associated ADR and PDR mutations were K103, V106, and V179, which cause high-level resistance to NVP and EFV (47). This is different from previous analyses of HIV-1 drug resistance in newly diagnosed HIV-infected patients in Sichuan Province (V106 and E138) (3) and the overall situation in China (K103 and Y181) (34). The study also found that FTC and 3TC were the most critical NRTI drugs responsible for high-level drug resistance, which is slightly different from other studies in China (ABC, FTC, and 3TC) (40). This shows the specificity of HIV DRM in Liangshan Prefecture and needs more attention in future clinical decision-making regarding ART regimens. The prevalence of the M184 mutation was significant in treatment failure patients and present at a lower level in newly diagnosed individuals, consistent with other observations. This may also pose a risk to the future use of a long-acting regimen combination of the capsid maturation inhibitor lenacapavir with NRTTI islatravir (48, 49). Our report suggests a higher prevalence of K65 mutations, which confer intermediate to high-level TFV and ABC resistance (50). This is higher than observed in high-income settings (51) and South Africa (48). The main ADR and PDR for PI included Q58 and M64 mutations. Resistance to PI was rare, which correlates with the late introduction of PIs into southwest China, the short duration of clinical application, and the high resistance barrier (19).

Our findings highlight age <18 years, injecting drug use, and initiation on NNRTIs-based regimen as independent risk factors for HIV ADR development. A previous study showed that HIV-infected children and adolescents exhibit markedly reduced virological suppression compared to adults (52). For PLWH under the age of 18 in Liangshan Prefecture, it is crucial to focus on the effectiveness of ART while paying special attention to drug resistance. Medications should be adjusted promptly for HIV-infected children and adolescents who experience poor treatment outcomes. Among the current PLWH in Liangshan Prefecture, injecting drug users represent a significant proportion. Due to the high rates of drug resistance in this group, it is essential to enhance the monitoring of treatment effectiveness. Additionally, the risks associated with initial regimens based on NNRTIs suggest that ART regimens with higher genetic barriers to drug resistance should be selected as first-line therapy.

Different HIV-1 genotypes have different propensities to develop drug resistance and exhibit variable DRM profiles (38). We found minor variants as a risk factor for PDR, and CRF01_AE had a higher risk than CRF07_BC for NRTI PDR. Due to the trend of the proportion of CRF07_BC gradually decreasing and other HIV-1 variants increasing in newly diagnosed individuals, the findings emphasize the importance of continuous monitoring of HIV subtypes and resistance patterns, especially in the newly diagnosed group. Additionally, the incidence of NNRTI PDR was significantly higher in newly diagnosed individuals with recent CD4 T-cell counts <200/μl than in the >350/μl group. This highlights the importance of improving HIV test coverage to reduce late diagnoses. For future clinical treatment plans for newly diagnosed individuals, it is recommended to consider the results of genotypic and drug resistance testing.

The slight decrease in ADR over the last three years may be linked to the increased focus and strategic changes made recently, further highlighting that improving management and prioritizing drug resistance can lead to lower resistance rates. The increase in PDR indicates that our future efforts to address drug resistance should extend beyond just the treatment population. It is essential to focus more on newly diagnosed individuals and enhance pre-treatment drug resistance testing. Over the past three years (10), changes in resistance to NNRTIs, NRTIs, and PIs in Liangshan Prefecture have shown a relatively slow increase. However, there is a general upward trend that aligns with recent global patterns. This situation underscores the serious challenges posed by drug resistance and highlights the urgent need to bolster research and control efforts.

Our study has several key strengths. First, blood samples of all treatment failure patients and most newly diagnosed individuals were collected and analyzed from all counties and cities in Liangshan Prefecture. Second, the study was conducted from 2021 to 2023 and provides a recent and comprehensive overview of both ADR and PDR in Liangshan Prefecture. Third, our sample population’s characteristics and proportions closely align with the overall PLWH population in the region, making it representative of the situation in Liangshan Prefecture.

Our study has some limitations. First, as a cross-sectional survey, this study only captured resistance at a single time point rather than longitudinally during an infection course. Second, our study lacked information on ART treatment adherence, which prevented us from considering the impact of adherence on drug resistance. Third, the variety of treatment regimens included in our sample is too extensive, which limits our ability to perform direct comparisons between the regimens.

## 5 Conclusion

In conclusion, we reported the prevalence of ADR and PDR for the first time in Liangshan Prefecture. While current therapies remain largely effective, the high level of PDR requires ongoing surveillance and possibly adjustments in treatment protocols. It is necessary to pay attention to the obstacle caused by PDR in the prevention and control of HIV epidemics.

## Data Availability

The datasets presented in this article are not readily available due to privacy or ethical restrictions. Requests to access the datasets should be directed to rongfry@163.com.

## Footnotes

^1^ https://hivdb.stanford.edu/hivdb/by-sequences/

^2^ http://www.hiv.lanl.gov
